# Maternal microbiota shapes brain and neuroendocrine development in piglets

**DOI:** 10.1002/imt2.70182

**Published:** 2026-10-26

**Authors:** Leli Wang, Chao Yan, Lei Li, Yuankun Deng, Hua Shao, Kuo Long, Cimin Long, Yuanyuan Zhu, Meiqing Chen, Jialu Huang, Jinghua Jiang, Ziyi Lin, Yunying Yao, Tao Yang, Wenbin Guo, Liangpeng Ge, Jing Sun, Yuchun Ding, Zuohua Liu, Mingzhu Yin, Yulong Yin

**Affiliations:** ^1^ Institute of Subtropical Agriculture Chinese Academy of Sciences Changsha China; ^2^ College of Animal Science and Technology Hunan Agricultural University Changsha China; ^3^ Shenzhen Branch, Guangdong Laboratory of Lingnan Modern Agriculture, Key Laboratory of Livestock and Poultry Multi‐omics of MARA, Agricultural Genomics Institute at Shenzhen Chinese Academy of Agricultural Sciences Shenzhen China; ^4^ Faculty of Life and Health Sciences Shenzhen University of Advanced Technology Shenzhen China; ^5^ Clinical Research Center, Medical Pathology Center, Cancer Early Detection and Treatment Center and Translational Medicine Research Center Chongqing University Three Gorges Hospital, Chongqing University Chongqing China; ^6^ Hunan Institute of Animal and Veterinary Science Changsha China; ^7^ Institute of Medicinal Plant Development Beijing China; ^8^ Huazhi Biotechnology Co. Ltd Changsha China; ^9^ National Center of Technology Innovation for Pigs, Chongqing Academy of Animal Sciences, Key Laboratory of Pig Industry Science Ministry of Agriculture Chongqing China

**Keywords:** brain, germ free, HPA axis, maternal microbiota, piglet

## Abstract

The maternal microbiota plays a critical role in early‐life development, yet how maternal microbiota exposure shapes cellular and neuroendocrine programs during mammalian development remains poorly understood. Here, leveraging germ‐free (GF) and maternal microbiota transplantation (MMT) models, we generated an integrated multi‐organ porcine atlas by combining multi‐regional single‐nucleus RNA sequencing, spatial transcriptomics, bulk RNA sequencing of intestinal tissues, and metabolomics. Across five brain regions and the hypothalamic–pituitary–adrenal (HPA) axis, we profiled 673,623 high‐quality nuclei and revealed microbiota‐dependent remodeling of neuronal and glial states across early postnatal developmental stages. MMT reshaped neuronal maturation programs, altered the distribution of immature neuronal states, and elicited region‐specific transcriptional responses in glutamatergic and GABAergic neurons. MMT also reorganized neuron–glia interaction networks, accompanied by trends toward increased plasma glutamic acid and glutamine levels. Across the gut–brain–neuroendocrine axis, MMT was associated with coordinated remodeling of HPA‐axis cellular communication, stress‐response transcriptional programs, and immune signatures, together with reduced circulating cortisol levels. Integrated intestinal analyses revealed segment‐specific transcriptional and metabolic remodeling, including alterations in tryptophan/indole‐related metabolites and immune‐associated pathways. Cross‐species stratified linkage disequilibrium score regression further identified enrichment of human cognitive and neuropsychiatric trait‐associated genetic signals within cell‐type‐specific gene signatures derived from microbiota‐responsive porcine cell populations. Our study establishes a large‐animal cellular atlas linking maternal microbiota‐responsive remodeling across the brain, gut, and neuroendocrine axis, providing a translational framework for understanding how early‐life microbial signals shape mammalian developmental physiology.

## Highlights

- An integrated multi‐organ atlas profiled 673,623 nuclei across the porcine brain and hypothalamic–pituitary–adrenal (HPA) axis and incorporated spatial, intestinal transcriptomic, and metabolomic data.
- Maternal microbiota transplantation was associated with region‐specific neuronal and glial remodeling, altered neuron–glia communication, and trends toward increased circulating glutamic acid and glutamine levels.
- Maternal microbiota transplantation remodeled HPA‐axis stress‐response and immune programs with reduced cortisol, alongside segment‐specific intestinal and tryptophan‐related metabolic changes.
- Cross‐species analyses revealed enrichment of human cognitive and neuropsychiatric trait‐associated genetic signals in microbiota‐responsive porcine cell populations.

## Graphical abstract

Using germ‐free and maternal microbiota‐transplanted piglets, we constructed an integrated multi‐organ atlas combining snRNA‐seq, Stereo‐seq, intestinal transcriptomics, and metabolomics. Maternal microbiota transplantation was associated with neuronal and glial remodeling, altered neuron–glia communication involving NRG1/3–ERBB4 signaling, trends toward increased plasma glutamic acid and glutamine, segment‐specific intestinal metabolic remodeling, and reduced plasma cortisol. Cross‐species analyses further revealed enrichment and spatial localization of human cognitive and neuropsychiatric trait‐associated genetic signals in the porcine brain.

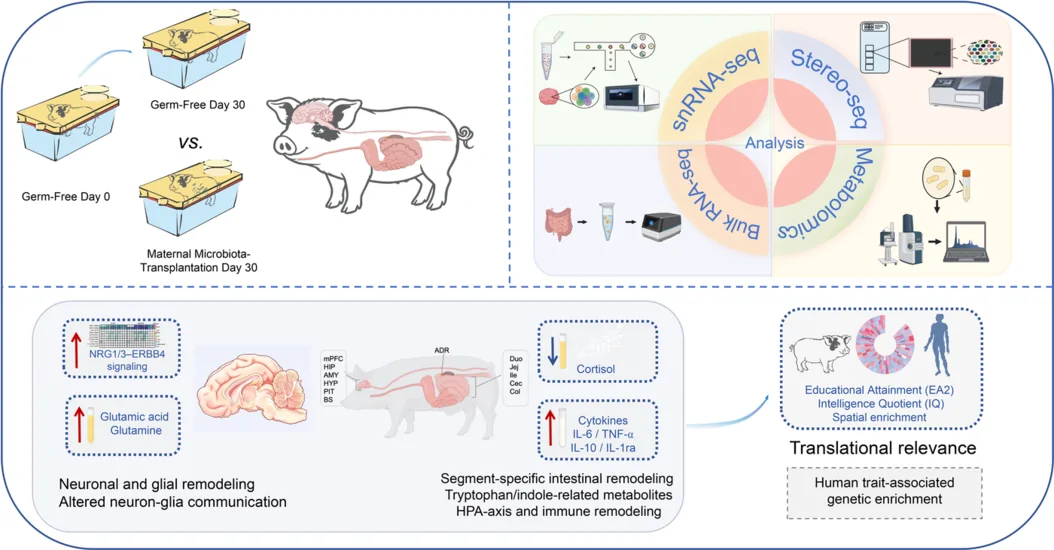

## INTRODUCTION

The mammalian brain is one of the most sophisticated organs, characterized by its extraordinary cellular heterogeneity and intricate functional organization [1]. Understanding its developmental trajectories and physiological regulation is essential for deciphering the mechanisms underlying brain maturation and neurological disorders. While rodent models have provided many insights, the search for translationally superior models has increasingly focused on the domestic pig (*Sus scrofa domesticus*) [2, 3, 4]. The porcine brain shares striking similarities with the human brain, including a gyrencephalic cortex, extensive basal‐thalamic‐cortical loops, and the circle of Willis, closely paralleling human neuroanatomy. White matter content in mature pigs and humans is similar high (>60%), compared with <12% in mature rats [5]. Notably, the global expression profiles of key regions, such as the cerebellum and hypothalamus, are more closely conserved between humans and pigs than between humans and mice [6]. Together with advances in genetic manipulation and multi‐omics technologies [7, 8], pigs provide a powerful large‐animal platform for investigating developmental neuroscience and the ways in which environmental factors shape physiological traits.

Recent multi‐omics studies integrating anatomical, transcriptomic, epigenomic, and spatial profiling have generated cell‐type resolved atlases for the porcine brain [9]. These studies have characterized multiple brain regions and identified cell type‐specific transcriptional and epigenomic programs, providing valuable insights into porcine brain organization and evolution [10, 11, 12, 13, 14]. Recent studies have uncovered cell‐type‐specific epigenomic regulatory mechanisms in the pig brain and examined the evolutionary dynamics between domestic pigs and wild boars, particularly in the cerebral cortex and cerebellum [15]. Comparative single‐cell analyses have revealed distinct regulatory programs differentiating domestic pigs from wild boars, whereas large‐scale spatial transcriptomic profiling across multiple brain regions identified the widespread distribution of microglia and the evolutionarily conserved transcription factor *MEF2C* as a key regulon [12]. However, brain development is not solely governed by intrinsic genetic programs but is continuously shaped by early‐life environmental cues [16].

The gut‐brain axis has emerged as a central bidirectional communication network linking intestinal homeostasis with brain development, physiology, and disease [17, 18]. Emerging evidence demonstrates that gut microbiota reshapes cellular composition, transcriptional programs, and neural function across multiple brain regions through diverse microbial metabolites, such as indole derivatives and short‐chain fatty acids [19], which influence host signaling pathways. For instance, *Lactobacillus murinus* selectively rescued social deficits in autism spectrum disorders (ASD) models while restoring cell type‐specific transcriptional programs in both the brain and intestine [20]. Maternal microbiota also plays a pivotal role in programming offspring development [21]. Previous studies have demonstrated that maternal microbiota regulates stem cell function in offspring [22] and influences fetal neurodevelopment during gestation [23], highlighting maternal microbiota exposure as a critical determinant of early‐life developmental trajectories. The neuroendocrine system, particularly the hypothalamic–pituitary–adrenal (HPA) axis, represents a key interface through which early‐life environmental signals may influence physiological adaptation and developmental trajectories. However, whether maternal microbiota exposure can shape the cellular and transcriptional organization of the developing brain and neuroendocrine system in a large mammal remains unknown.

Here, by integrating single‐nucleus RNA sequencing with spatial transcriptomics, we generated a high‐resolution multi‐regional atlas encompassing five major brain regions, the HPA axis, and five intestinal segments. We found that maternal microbiota transplantation remodels cellular composition, cell type‐specific transcriptional programs, and spatial organization across brain regions and the HPA axis. Together, these findings establish the pig as a powerful translational model for dissecting microbiota‐dependent developmental programming of the brain and neuroendocrine system, providing a framework for investigating gut‐brain interactions in large mammals and advancing our understanding of microbiota‐mediated developmental programming.

## RESULTS

### A maternal microbiota‐responsive cellular atlas reveals neurodevelopmental remodeling in the developing porcine brain

To elucidate how maternal microbiota exposure influences cellular states and transcriptional programs across the developing porcine brain, we constructed a high‐resolution multi‐regional single‐nucleus RNA sequencing (snRNA‐seq) atlas using GF piglets at Day 0 (GFD0), GF piglets at Day 30 (GFD30), and maternal microbiota‐transplanted piglets at Day 30 (MMTD30) (Figure 1A). After quality control and doublet removal, we retained 504,489 high‐quality nuclei from five neuroanatomical regions, including hypothalamus (HYP), brainstem (BS), amygdala (AMY), hippocampus (HIP), and medial prefrontal cortex (mPFC), spanning birth and early postnatal development (D0 and D30). Following batch correction and integration using Harmony [24], we identified six major cell lineages based on canonical marker genes [25] (Figure 1B,C; Table S1), including astrocytes (*AQP4* and *GFAP*), endothelial cells (*FLT1* and *VWF*), microglia (*CSF1R* and *CX3CR1*), neurons (*RBFOX1* and *NRXN3*), oligodendrocytes (*OPALIN* and *MOBP*), and oligodendrocyte precursor cells (OPCs) (*PCDH15* and *PDGFRA*). The identified major cell populations exhibited distinct transcriptional identities and were consistently detected across different neuroanatomical regions and experimental conditions (Figure S1A,B).

**FIGURE 1:**
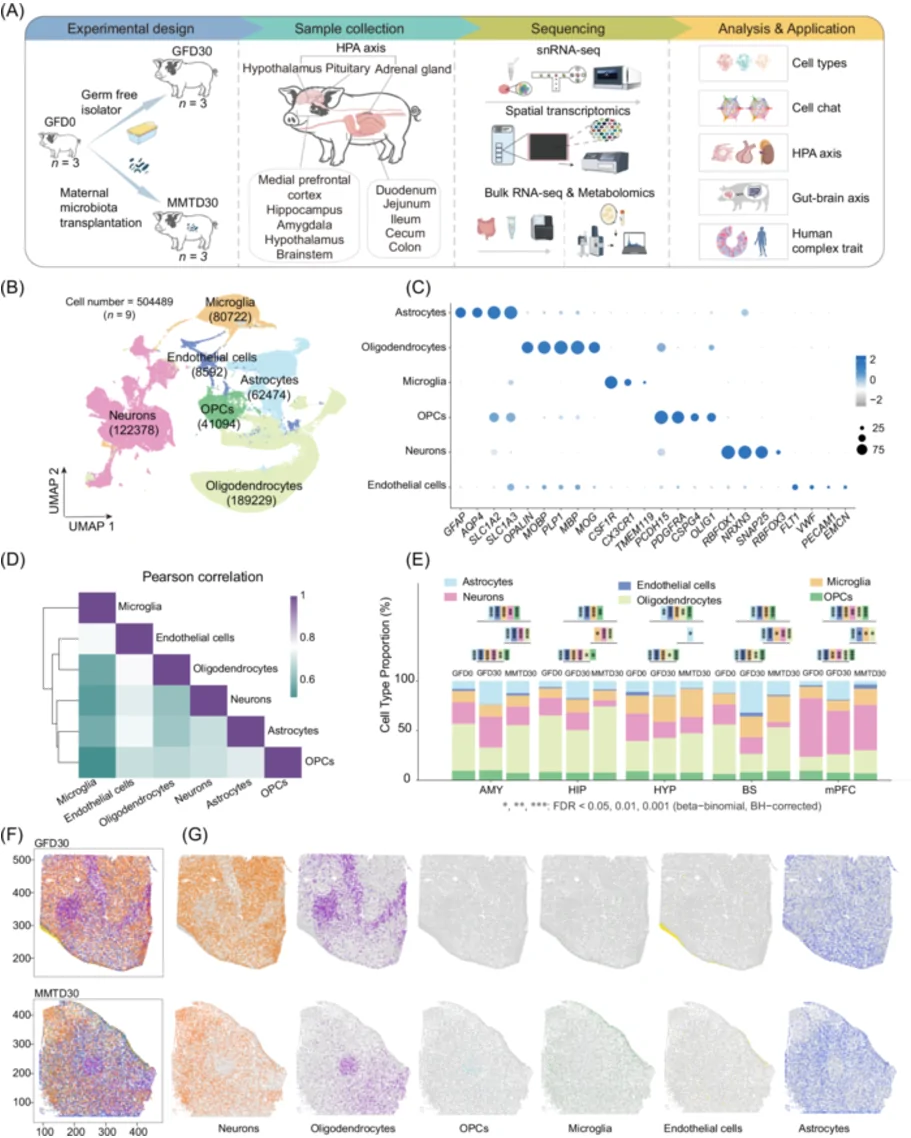
Multi‐regional single‐nucleus and spatial transcriptomic atlas of the porcine brain across developmental stages and microbial conditions. (A) Experimental design and analytical workflow. GFD0, GFD30, and MMTD30 piglets (*n* = 3 per group) were used for multi‐organ sample collection and downstream analyses, including five brain regions (AMY, HIP, HYP, BS, and mPFC), PIT, ADR, and five intestinal segments. (B) Integrated UMAP visualization of 504,489 high‐quality nuclei, colored by major cell type. Cell numbers are indicated for each population. (C) Dot plot of canonical marker genes for astrocytes, oligodendrocytes, microglia, OPCs, neurons, and endothelial cells. Dot size indicates the percentage of nuclei expressing each gene, and color indicates scaled average expression. (D) Hierarchically clustered pairwise correlation heatmap of transcriptional profiles across the six major cell lineages. (E) Relative proportions of the six major cell lineages across five brain regions and three experimental groups. (F) Composite spatial maps of the six major cell populations in the HYP sections from GFD30 and MMTD30 piglets. (G) Spatial distributions of individual major cell populations in the corresponding HYP sections. The upper and lower rows represent GFD30 and MMTD30, respectively. ADR, adrenal gland; AMY, amygdala; BS, brainstem; GFD0, germ‐free piglets at postnatal Day 0; GFD30, germ‐free piglets at postnatal Day 30; HIP, hippocampus; HYP, hypothalamus; MMTD30, maternal microbiota‐transplanted piglets at postnatal Day 30; mPFC, medial prefrontal cortex; OPCs, oligodendrocyte precursor cells; PIT, pituitary.

We next examined the transcriptional relationships and compositional characteristics of the six major cell populations. Pearson correlation analysis based on average gene‐expression profiles revealed distinct transcriptional relationships among neuronal and glial populations (Figure 1D). Their relative abundances also varied across brain regions, developmental stages, and microbial conditions, highlighting cellular heterogeneity across the developing porcine brain (Figure 1E). For example, neurons showed increased relative proportions in the AMY and HIP of GFD30 group than in MMTD30 group. In addition, oligodendrocytes were increased, while astrocytes were reduced in MMTD30 compared with GFD30, suggesting that maternal microbiota is associated with selective remodeling of neuronal–glial composition during early postnatal development. To further characterize the functional properties associated with each major cell population, cell‐type marker genes displayed distinct expression patterns corresponding to six cell‐type‐specific clusters (C1–C6; Figure S1C). The enriched functional categories were consistent with established physiological roles of each cell population. For instance, neurons were significantly enriched for synaptic communication‐related processes, including “chemical synaptic transmission,” and “anterograde trans‐synaptic signaling.” Oligodendrocytes were predominantly associated with “regulation of oligodendrocyte differentiation,” “regulation of glial cell differentiation,” and “oligodendrocyte differentiation.”

High‐resolution spatial transcriptomic profiles of the HYP (Figure 1F,G; Figure S2A) were generated using Stereo‐seq to examine the spatial organization of major cell populations, with this region selected for its key role as a neuroendocrine hub linking gut microbial signals to peripheral physiology. Cell types identified from snRNA‐seq were mapped onto spatial sections using label transfer [25], enabling high‐confidence spatial annotation of cellular domains. Spatial mapping confirmed the regional distribution of major cell populations and revealed microbiota‐associated remodeling of cellular architecture within the HYP. The spatial transcriptomic profiles consistently captured the six major cell populations in both groups (Figure S2B), while comparison of their inferred relative proportions revealed microbiota‐associated alterations in cellular composition (Figure S2C). Together, these findings demonstrate that maternal microbiota exposure is associated with coordinated remodeling of cellular composition and spatial organization across the developing porcine brain.

### Maternal microbiota exposure remodels neuronal states and neuron–glia communication during early brain development

To investigate how maternal microbiota reshapes neuronal transcriptional programs, we first compared differential‐expression profiles across brain regions and cell types between MMTD30 and GFD30 piglets. Neurons exhibited the largest number of differentially expressed genes (DEGs) across multiple brain regions, indicating that neuronal populations represent major transcriptional responders to maternal microbiota exposure during early postnatal brain development (Figure 2A; Table S2). We next identified three major gene expression patterns among GFD0, GFD30, and MMTD30 piglets, corresponding to early developmental programs, postnatal maturation programs, and microbiota‐associated transcriptional states (Figure 2B).

**FIGURE 2:**
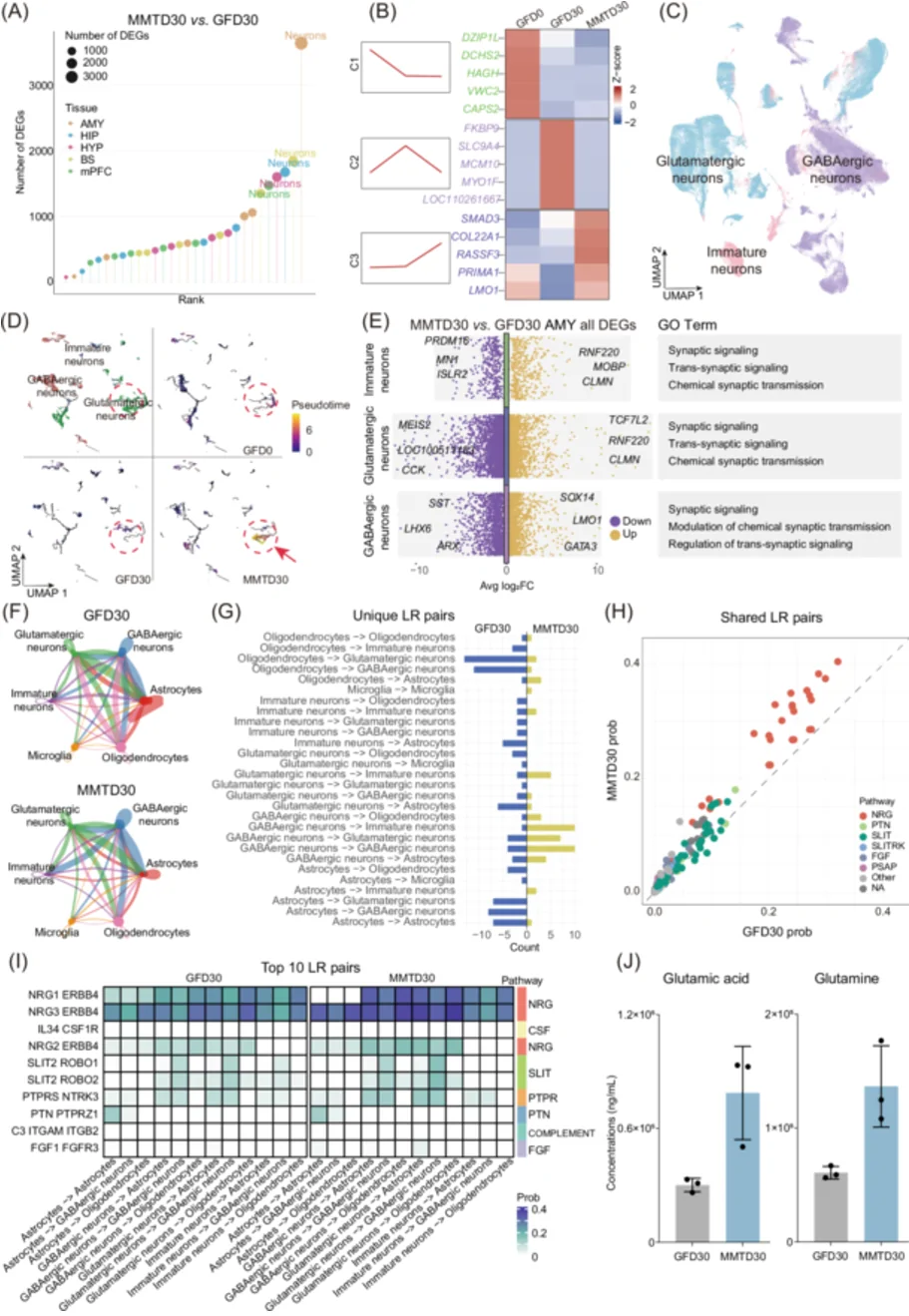
Maternal microbiota‐associated remodeling of neuronal transcriptional states, developmental trajectories, intercellular communication, and plasma glutamate–glutamine metabolism. (A) Ranked numbers of DEGs across cell types and five brain regions between MMTD30 and GFD30 piglets. Dot size indicates the number of DEGs, and colors denote brain regions. (B) Three gene‐expression patterns (C1–C3) across GFD0, GFD30, and MMTD30 identified using ClusterGVis. The heatmap shows scaled expression (Z‐score), and line plots summarize the corresponding group‐associated expression patterns. (C) UMAP visualization of neuronal nuclei classified into three major populations: glutamatergic neurons, GABAergic neurons, and immature neurons. (D) Pseudotime trajectory of neuronal populations. The integrated trajectory is colored by pseudotime, with group‐stratified distributions shown for GFD0, GFD30, and MMTD30. (E) Differential gene‐expression profiles of glutamatergic, GABAergic, and immature neurons in the AMY between MMTD30 and GFD30. Representative upregulated and downregulated genes are labeled, with selected enriched GO terms shown on the right. (F) CellChat‐inferred intercellular communication networks among astrocytes, GABAergic neurons, glutamatergic neurons, immature neurons, microglia, and oligodendrocytes in the GFD30 and MMTD30 piglets. (G) Numbers of group‐specific ligand–receptor pairs across source–target cell combinations. Blue and yellow bars indicate interactions detected only in the GFD30 and MMTD30, respectively. (H) Comparison of inferred communication probabilities for LR pairs shared between GFD30 and MMTD30. Colors denote signaling pathways, and the dashed diagonal indicates equal communication probability between groups. (I) Heatmaps of the top 10 LR pairs across selected source–target cell combinations in the GFD30 and MMTD30. Color indicates inferred communication probability. (J) Plasma concentrations of glutamic acid and glutamine in the GFD30 and MMTD30 piglets. Individual points represent independent biological replicates. Data are presented as mean ± SD. DEGs, differentially expressed genes; GO, Gene Ontology; GFD0, germ‐free piglets at postnatal Day 0; GFD30, germ‐free piglets at postnatal Day 30; LR, ligand–receptor; MMTD30, maternal microbiota‐transplanted piglets at postnatal Day 30. All snRNA‐seq and plasma analyses were based on three independent piglets per group (*n* = 3).

Neuronal populations were then isolated and classified into three major subtypes: glutamatergic neurons (*SLC17A7* and *CAMK2A*), GABAergic neurons (*GAD1* and *GAD2*), and immature neurons (*DCX* and *SOX11*) (Figures 2C, S3A). The relative proportions of these neuronal subtypes varied across brain regions and experimental groups (Figure S3B). Animal‐level comparisons further revealed brain‐region‐ and subtype‐specific differences in neuronal proportions between MMTD30 and GFD30 piglets (Figure S3C). Pseudotime analysis further arranged the three neuronal populations along an inferred developmental trajectory, with immature neurons occupying an early region of the trajectory and glutamatergic and GABAergic neurons distributed along subsequent states (Figures 2D, S3D). These findings suggest that maternal microbiota transplantation was associated with transcriptional signatures related to excitatory neuronal maturation during early postnatal brain development.

To investigate whether maternal microbiota transplantation was associated with altered neuronal subtype distribution, we performed ratio of observed to expected cell numbers (Ro/e) enrichment analysis [26]. Overall, GABAergic neurons were enriched in the BS and HYP (Figure S3E). We next examined brain‐region‐ and neuronal‐subtype‐specific transcriptional responses associated with maternal microbiota transplantation. Differential expression analysis identified a number of DEGs in immature, glutamatergic, and GABAergic neurons in the AMY (Figure 2E; Tables S2 and S3), HIP, HYP, BS, and mPFC (Figure S4A) between MMTD30 and GFD30 groups. In the AMY, DEGs from immature and glutamatergic neurons were enriched in synaptic signaling, trans‐synaptic signaling, and chemical synaptic transmission, whereas GABAergic neuronal DEGs were enriched in synaptic signaling, modulation of chemical synaptic transmission, and regulation of trans‐synaptic signaling.

To distinguish microbiota‐associated transcriptional differences from developmental changes occurring under GF conditions, we additionally compared GFD30 with GFD0, which demonstrated substantial developmental‐stage‐associated transcriptional differences among the three neuronal populations across all five brain regions (Figure S4B; Tables S4 and S5). Notably, *LMO1* exhibited a dynamic expression pattern, showing decreased expression from GFD0 to GFD30 but increased expression following maternal microbiota transplantation in MMTD30 compared with GFD30 piglets. This pattern suggests that maternal microbiota exposure may modulate transcriptional trajectories associated with early postnatal neuronal maturation. To provide histological validation of the GABAergic neuronal population identified by snRNA‐seq, we performed VGAT immunofluorescence staining in the HYP. VGAT‐positive GABAergic cells were observed in both GFD30 and MMTD30 piglets. Quantitative analysis showed a numerically higher number of VGAT‐positive cells in MMTD30 piglets, although the difference did not reach statistical significance (Figure S5).

Beyond the neurocentric paradigm, the functional integrity of the mammalian brain is increasingly recognized as a product of the intricate and indispensable crosstalk within the neuron–glia communication [27, 28]. CellChat analysis revealed extensive inferred communication among astrocytes, GABAergic neurons, glutamatergic neurons, immature neurons, microglia, and oligodendrocytes in both GFD30 and MMTD30 piglets (Figure 2F). Comparison of ligand–receptor interactions identified distinct sets of group‐specific interactions between the two conditions (Figure 2G). Among ligand–receptor pairs shared between GFD30 and MMTD30, inferred communication probabilities also differed substantially, involving signaling pathways including NRG, PTN, SLIT, SLITRK, FGF, and PSAP (Figure 2H). Notably, NRG‐associated shared interactions exhibited higher inferred communication probabilities in MMTD30 than in GFD30, including ligand–receptor pairs NRG1–ERBB4 and NRG3–ERBB4 (Figure 2I). The NRG1–ERBB4 and NRG3–ERBB4 axes have been implicated in neuronal maturation and synaptic development, suggesting a potential pathway through which maternal microbiota exposure may influence neural developmental programs. Region‐resolved CellChat analyses further demonstrated substantial heterogeneity across the AMY, HIP, HYP, BS, and mPFC between GFD30 and MMTD30 in a region‐dependent manner (Figure S6A). Given the altered glutamatergic transcriptional signatures, we next examined systemic metabolites related to glutamate neurotransmitter metabolism [29]. Plasma glutamic acid and glutamine showed increasing trends in MMTD30 compared with GFD30 piglets (Figure 2J), indicating that maternal microbiota transplantation was accompanied by alterations in the circulating glutamate–glutamine metabolic profile. Together, these findings indicate that maternal microbiota transplantation is associated not only with neuronal transcriptional remodeling but also with region‐specific reorganization of inferred neuron–glia communication networks.

### Maternal microbiota transplantation is associated with glial transcriptional states and regulatory programs during early brain development

To further characterize glial transcriptional and regulatory heterogeneity associated with maternal microbiota transplantation, we extracted astrocytes, microglia, and oligodendrocytes (Figure 3A). Ro/e enrichment analysis revealed brain‐region‐ and group‐dependent distributions of major brain cell populations across GFD0, GFD30, and MMTD30 piglets (Figure S7A). Single‐Cell rEgulatory Network Inference and Clustering (SCENIC) analysis revealed distinct regulatory programs across astrocytes, microglia, and oligodendrocytes. Regulon specificity analysis identified six co‐regulatory modules associated with trans‐synaptic signaling, neuronal development, and cell morphogenesis, and cell‐junction and synapse organization (Figures 3B, S7B).

**FIGURE 3:**
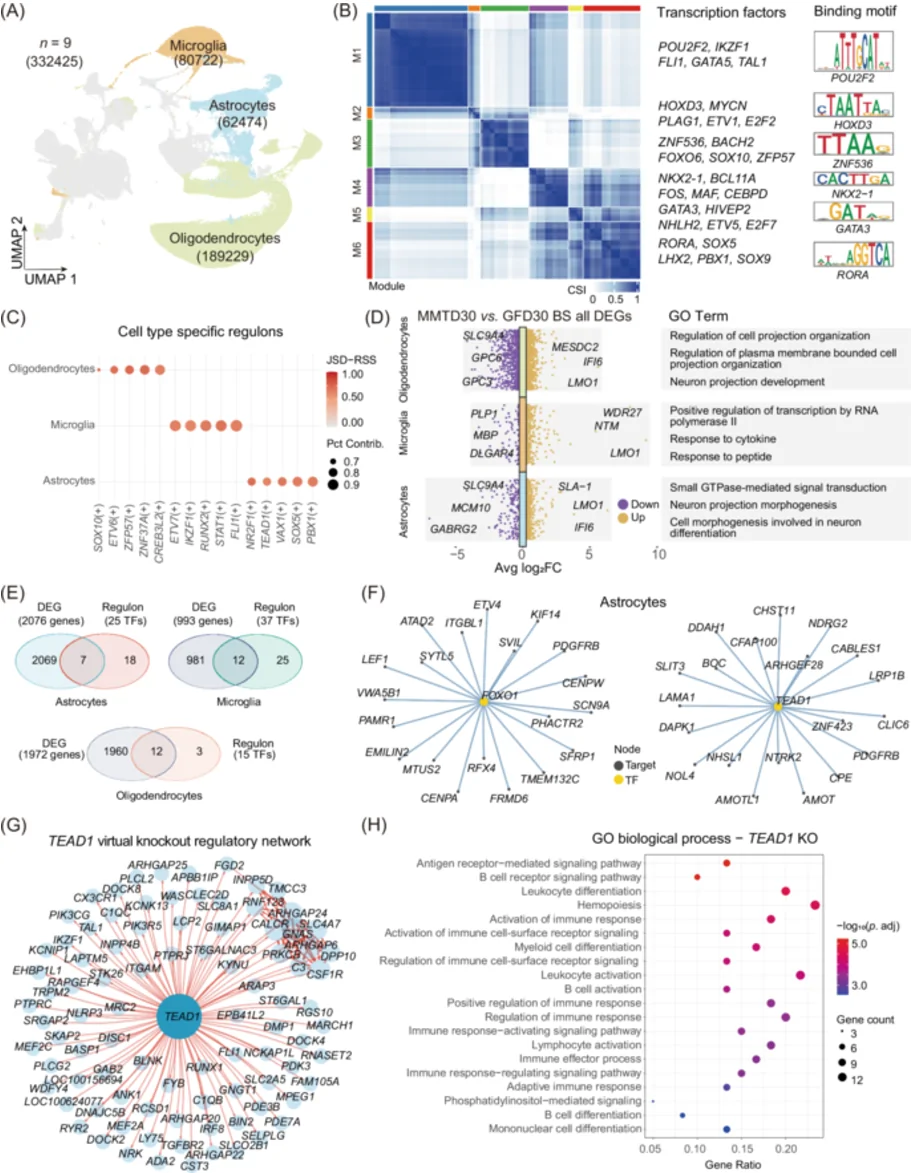
Transcriptional regulatory programs and candidate regulons in major glial populations of the porcine brain. (A) UMAP visualization of 332,425 glial nuclei, comprising astrocytes, microglia, and oligodendrocytes. Cell numbers are indicated. (B) CSI‐based analysis of SCENIC regulons showing six co‐regulatory modules (M1–M6). Representative transcription factors and corresponding binding motifs are shown for each module. Color intensity indicates the CSI value. (C) Cell‐type‐specific regulons identified using Jensen–Shannon divergence‐based regulon specificity scores (JSD‐RSS). Dot color indicates JSD‐RSS, and dot size indicates the contribution of each regulon. (D) Differential gene‐expression profiles of oligodendrocytes, microglia, and astrocytes in the BS between MMTD30 and GFD30 piglets. Representative genes and selected enriched GO terms are shown. (E) Overlap between cell‐type DEGs and SCENIC‐derived regulon transcription factors in astrocytes, microglia, and oligodendrocytes. (F) FOXO1‐ and TEAD1‐centered regulon networks and their predicted target genes in astrocytes. (G) Regulatory network of genes predicted to respond to *in silico TEAD1* knockout in MMTD30 astrocytes. (H) GO enrichment of genes predicted to respond to *TEAD1* knockout. Dot size indicates gene count, and color represents −log_10_(adjusted *p* value). CSI, connection specificity index; DEG, differentially expressed gene; GFD30, germ‐free piglets at postnatal Day 30; JSD, Jensen–Shannon divergence; MMTD30, maternal microbiota‐transplanted piglets at postnatal Day 30; TF, transcription factor; RSS, regulon specificity score. All snRNA‐seq analyses were based on three independent piglets per experimental group (*n* = 3); individual nuclei were not treated as independent biological replicates.

Regulon specificity analysis further identified distinct cell‐type‐specific transcriptional regulators among the three major glial populations (Figure 3C), including SOX10‐associated oligodendrocyte programs, RUNX2‐associated microglial regulatory programs, and TEAD1‐associated astrocytic regulatory programs. These cell‐type‐specific regulon profiles revealed distinct regulatory architectures underlying glial identity and function. Animal‐level comparisons revealed region‐ and cell‐type‐specific differences in the proportions of astrocytes, microglia, and oligodendrocytes between MMTD30 and GFD30 piglets (Figure S7C). Ro/e enrichment analysis further revealed region‐ and group‐dependent distribution patterns of these glial populations across the five brain regions and three experimental conditions (Figure S7D).

We next examined region‐ and cell‐type‐specific transcriptional responses of the three glial populations. In the BS, comparison of MMTD30 with GFD30 revealed distinct differential‐expression profiles in oligodendrocytes, microglia, and astrocytes (Figure 3D). Oligodendrocyte‐associated DEGs were enriched in the regulation of cell‐projection organization and neuron‐projection development; microglial DEGs were associated with positive regulation of transcription by RNA polymerase II and responses to cytokines and peptides; whereas astrocyte‐associated DEGs were enriched in neuron‐projection morphogenesis, and cell morphogenesis involved in neuronal differentiation. Parallel analyses in the AMY, HIP, HYP, and mPFC likewise revealed region‐ and cell‐type‐dependent differential‐expression profiles between MMTD30 and GFD30 (Figure S7E). To distinguish these microbiota‐associated differences from transcriptional changes accompanying postnatal development under GF conditions, we additionally compared GFD30 with GFD0. Astrocytes, microglia, and oligodendrocytes exhibited distinct developmental‐stage‐associated DEG profiles across the AMY, HIP, HYP, BS, and mPFC (Figure S8). These findings indicate that maternal microbiota‐associated glial responses are shaped by both cellular identity and anatomical context. To further validate microglial alterations at the histological level, we performed IBA1 immunofluorescence staining in the HYP. GFD30 piglets exhibited significantly higher mean IBA1 fluorescence intensity than MMTD30 piglets, whereas the difference in IBA1‐positive microglial soma area did not reach statistical significance (Figure S9).

Given the prominent regulatory heterogeneity of astrocytes, we further resolved this population into six transcriptional subclusters (A1–A6). These states displayed distinct functional programs, including glial differentiation, cAMP/PKA signaling, metabolic regulation, synaptic modulation, and cell migration (Figure S10A,B). These findings highlight substantial transcriptional heterogeneity and plasticity among astrocytes associated with maternal microbiota transplantation. Pseudotime analysis further revealed a continuous distribution of the six astrocyte subclusters (A1–A6) across distinct transcriptional states, accompanied by marked differences in their group‐specific distributions along pseudotime (Figure S10C,D). Together, these results suggest that maternal microbiota transplantation is associated with coordinated remodeling of astrocyte transcriptional‐state composition during early postnatal development. Moreover, MMTD30 versus GFD30 comparisons revealed subcluster‐ and region‐specific transcriptional responses across all five brain regions (Figure S11).

To prioritize transcription factors potentially associated with glial transcriptional remodeling, we integrated cell‐type‐specific DEGs with SCENIC‐derived regulon transcription factors. Astrocytes contained 2076 DEGs and 25 regulon‐associated transcription factors, with 7 overlapping factors; microglia contained 993 DEGs and 37 regulon‐associated transcription factors, with 12 overlapping factors; and oligodendrocytes contained 1972 DEGs and 15 regulon‐associated transcription factors, also with 12 overlapping factors (Figure 3E). Regulon‐network visualization further highlighted FOXO1‐ and TEAD1‐centered regulatory architectures and their predicted target genes in astrocytes (Figure 3F). Among these candidate regulators, TEAD1, and FOXO1 emerged as prominent regulatory nodes in astrocytes, motivating downstream in silico perturbation analyses.

To further explore the potential regulatory consequences of these candidate transcription factors, we performed in silico knockout analysis using scTenifoldKnk [30]. Virtual perturbation of *TEAD1* predicted extensive downstream regulatory changes involving genes such as *CSF1R*, *C3*, *INPP5D*, *ITGAM*, *CX3CR1*, and *NLRP3* (Figure 3G). Functional enrichment of the predicted TEAD1‐responsive genes was dominated by immune‐related processes, including regulation and activation of immune responses, leukocyte activation and differentiation, myeloid cell differentiation, and immune receptor signaling (Figure 3H). In parallel, virtual *FOXO1* knockout generated a distinct predicted regulatory network (Figure S12A), with responsive genes predominantly enriched in cilium organization and assembly, axoneme assembly, microtubule‐based movement, and cilium‐dependent motility‐related processes (Figure S12B). An additional virtual perturbation of *LMO1* also predicted extensive downstream regulatory changes (Figure S12C). In contrast to *FOXO1*, genes predicted to respond to *LMO1* knockout were predominantly enriched in immune‐related processes, including adaptive immune response, immune response‐regulating and activating signaling pathways, leukocyte activation and differentiation, myeloid cell differentiation, cytokine production, and B‐cell receptor signaling (Figure S12D). Thus, the *in silico* perturbation analyses predicted distinct downstream functional programs associated with candidate transcriptional regulators. Collectively, these findings reveal pronounced cell‐type‐ and region‐specific glial regulatory remodeling associated with maternal microbiota transplantation, uncover substantial heterogeneity of astrocyte transcriptional states, and nominate TEAD1 as a candidate regulator associated with astrocytic transcriptional programs during early brain development.

### Maternal microbiota transplantation reshapes HPA axis communication and stress‐related transcriptional programs during early development

To characterize maternal microbiota‐associated remodeling of the HPA axis, we analyzed snRNA‐seq profiles from the HYP, pituitary (PIT), and adrenal gland (ADR). Cell‐type annotation revealed distinct tissue‐specific cellular landscapes, including neuronal and glial populations in the HYP, diverse endocrine and supporting cell populations in the PIT, and adrenocortical, chromaffin, vascular, and immune populations in the ADR (Figure 4A). The relative proportions of cell populations varied across GFD0, GFD30, and MMTD30 piglets within each HPA‐axis tissue (Figure S13A). These results established a cellular framework for dissecting microbiota‐associated neuroendocrine remodeling across the HPA axis.

**FIGURE 4:**
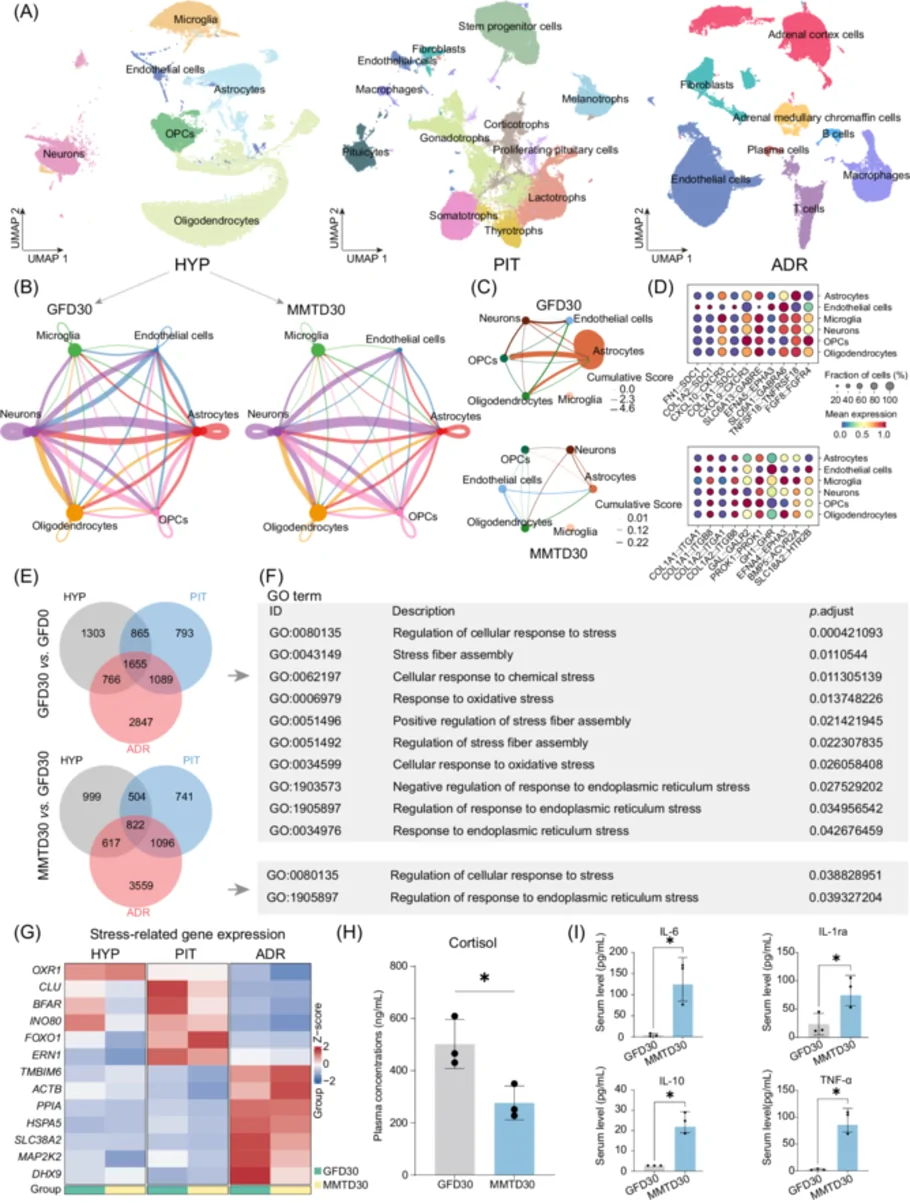
Maternal microbiota transplantation is associated with HPA‐axis communication, stress‐related transcriptional remodeling, and peripheral immune alterations. (A) UMAP visualizations of nuclei from the HYP, PIT, and ADR, colored by annotated cell type. (B) CellChat‐inferred intercellular communication networks among major HYP cell populations in GFD30 and MMTD30 piglets. Edge widths indicate the number of inferred interactions. (C) Cumulative‐score interaction networks among major HYP cell populations. The upper and lower networks represent GFD30 and MMTD30 piglets. Edge widths indicate the cumulative interaction score. (D) Dot plots showing expression patterns of selected ligand–receptor gene pairs across HYP cell populations in GFD30 (upper) and MMTD30 (lower) piglets. Dot size indicates the fraction of cells expressing the corresponding genes, and color indicates mean expression within the group. (E) Venn diagrams showing shared and tissue‐specific DEGs among the HYP, PIT, and ADR under the GFD30 versus GFD0 comparison (upper) and the MMTD30 versus GFD30 comparison (lower). (F) GO enrichment of the 1655 DEGs shared across the HYP, PIT, and ADR in the GFD30 versus GFD0 comparison (upper) and the 822 shared DEGs in the MMTD30 versus GFD30 comparison (lower). Adjusted *p* values are shown. (G) Heatmap of representative stress‐related genes across the HYP, PIT, and ADR in GFD30 and MMTD30. Colors indicate row‐scaled *Z*‐scores. (H) Plasma cortisol concentrations in GFD30 and MMTD30 piglets. Data are presented as mean ± SD. *p < 0.05. (I) Serum concentrations of IL‐6, IL‐1ra, IL‐10, TNF‐α in GFD30 and MMTD30 piglets. Individual points represent independent biological replicates. Data are presented as mean ± SD. *p < 0.05. ADR, adrenal gland; DEG, differentially expressed gene; GO, Gene Ontology; GFD0, germ‐free piglets at postnatal Day 0; GFD30, germ‐free piglets at postnatal Day 30; HPA, hypothalamic–pituitary–adrenal; HYP, hypothalamus; MMTD30, maternal microbiota‐transplanted piglets at postnatal Day 30; OPCs, oligodendrocyte precursor cells; PIT, pituitary. All group‐level analyses were based on three independent piglets per group (*n* = 3).

CellChat analysis revealed extensive inferred communication networks across the HYP, PIT, and ADR, with MMTD30‐associated alterations in interaction strength and ligand–receptor usage. In the HYP, these alterations involved neuronal, glial, and endothelial populations between GFD30 and MMTD30 piglets, with group‐dependent differences in network architecture and interaction patterns (Figure 4B). Cumulative‐score analysis further highlighted differences in the overall configuration of inferred communication among these HYP cell populations (Figure 4C), as well as ligand–receptor expression patterns that differed between GFD30 and MMTD30 piglets, with distinct interaction profiles observed across HYP cell populations in the spatial transcriptomic dataset (Figure 4D). Consistent with these observations, CellChat analyses in the PIT and ADR likewise revealed group‐associated differences in inferred intercellular communication networks between GFD30 and MMTD30 piglets (Figure S13B,C). These analyses indicate that maternal microbiota transplantation is associated with tissue‐specific reorganization of inferred cell–cell communication across the HPA axis.

To further resolve transcriptional changes at cell‐type resolution, we compared differential‐expression profiles across major cell populations in the HYP, PIT, and ADR (Figure S14A). The MMTD30 versus GFD30 comparison revealed heterogeneous transcriptional responses among neuronal and glial populations in the HYP, endocrine, and supporting populations in the PIT, and endocrine, vascular, stromal, and immune populations in the ADR. A parallel comparison of GFD30 with GFD0 similarly revealed pronounced cell‐type‐specific transcriptional differences across all three tissues (Figure S14B). Thus, both postnatal development under GF conditions and maternal microbiota transplantation were associated with highly cell‐type‐dependent transcriptional responses across the HPA axis.

To further define transcriptional remodeling across the HPA axis, we compared DEGs shared among the HYP, PIT, and ADR. Under the GFD30 versus GFD0 comparison, 1655 DEGs were shared across all three tissues, whereas 1303, 793, and 2847 DEGs were specific to the HYP, PIT, and ADR, respectively (Figure 4E). In comparison, the MMTD30 versus GFD30 analysis identified 822 DEGs shared across the three HPA‐axis tissues, together with 999 HYP‐specific, 741 PIT‐specific, and 3559 ADR‐specific DEGs. These findings reveal a coordinated transcriptional architecture across the HPA axis, characterized by conserved stress‐response programs and tissue‐specific regulatory responses associated with maternal microbiota transplantation. Functional enrichment analysis further revealed a prominent stress‐response signature among the shared transcriptional changes (Figure 4F). The 1655 DEGs shared across the HYP, PIT, and ADR in the GFD30 versus GFD0 comparison were enriched in biological processes including regulation of cellular response to stress, cellular response to chemical and oxidative stress, stress fiber assembly, and regulation of endoplasmic reticulum stress. The 822 shared DEGs identified in the MMTD30 versus GFD30 comparison were also enriched in the regulation of cellular response to stress and the regulation of endoplasmic reticulum stress. Thus, these results identify stress‐response regulation as a conserved transcriptional signature across the HPA axis.

To identify coordinated regulatory programs across the HPA axis, we examined transcriptional changes shared among the HYP, PIT, and ADR. Consistent with the functional enrichment results, representative stress‐related genes selected from the shared HPA‐axis DEG set, including OXR1, CLU, BFAR, INO80, FOXO1, ERN1, TMBIM6, ACTB, PPIA, HSPA5, SLC38A2, MAP2K2, and DHX9, displayed distinct tissue‐ and group‐dependent expression patterns across the HYP, PIT, and ADR (Figures 4G, S14C). At the physiological level, plasma cortisol concentrations were significantly lower in MMTD30 than in GFD30 piglets (*p* < 0.05; Figure 4H), consistent with altered HPA‐axis activity following maternal microbiota transplantation. To further determine whether these neuroendocrine changes were accompanied by alterations in peripheral immune status, we measured representative serum cytokines. Compared with GFD30 piglets, MMTD30 piglets exhibited significantly higher serum IL‐6 and IL‐1ra concentrations (*p* < 0.05), together with significant increases in IL‐10 and TNF‐α (*p* < 0.05; Figure 4I), suggesting a complex immune‐state remodeling rather than a uniformly pro‐inflammatory or anti‐inflammatory shift. Collectively, these findings demonstrate that maternal microbiota transplantation is associated with coordinated remodeling of HPA‐axis cellular communication, conserved stress‐response transcriptional programs, and systemic neuroendocrine–immune states during early development.

### Maternal microbiota transplantation remodels regional intestinal transcriptional and metabolic landscapes during early development

To characterize intestinal transcriptional responses associated with maternal microbiota transplantation, we analyzed transcriptomic profiles across five intestinal segments, including the duodenum, jejunum, ileum, cecum, and colon (Figure 5A). Weighted gene co‐expression network analysis (WGCNA) revealed distinct module eigengene expression patterns across intestinal segments and experimental groups (Figure 5B). Module–trait analysis further revealed pronounced associations between intestinal transcriptional modules and microbial status or developmental stage (Figure 5C). MEgreenyellow and MEred showed the strongest positive correlations with microbial status (*r* = 0.79, *p* = 7 × 10^−11^; *r* = 0.71, *p* = 5 × 10^−8^, respectively), whereas MEsalmon was negatively associated with both microbial status (*r* = −0.61, *p* = 1 × 10^−5^) and Day30 (*r* = −0.72, *p* = 3 × 10^−8^). Consistent with these results, principal component analysis showed pronounced segment‐dependent transcriptional variation across the intestinal tract (Figure S15A), highlighting strong regional specialization of intestinal responses to maternal microbiota exposure.

**FIGURE 5:**
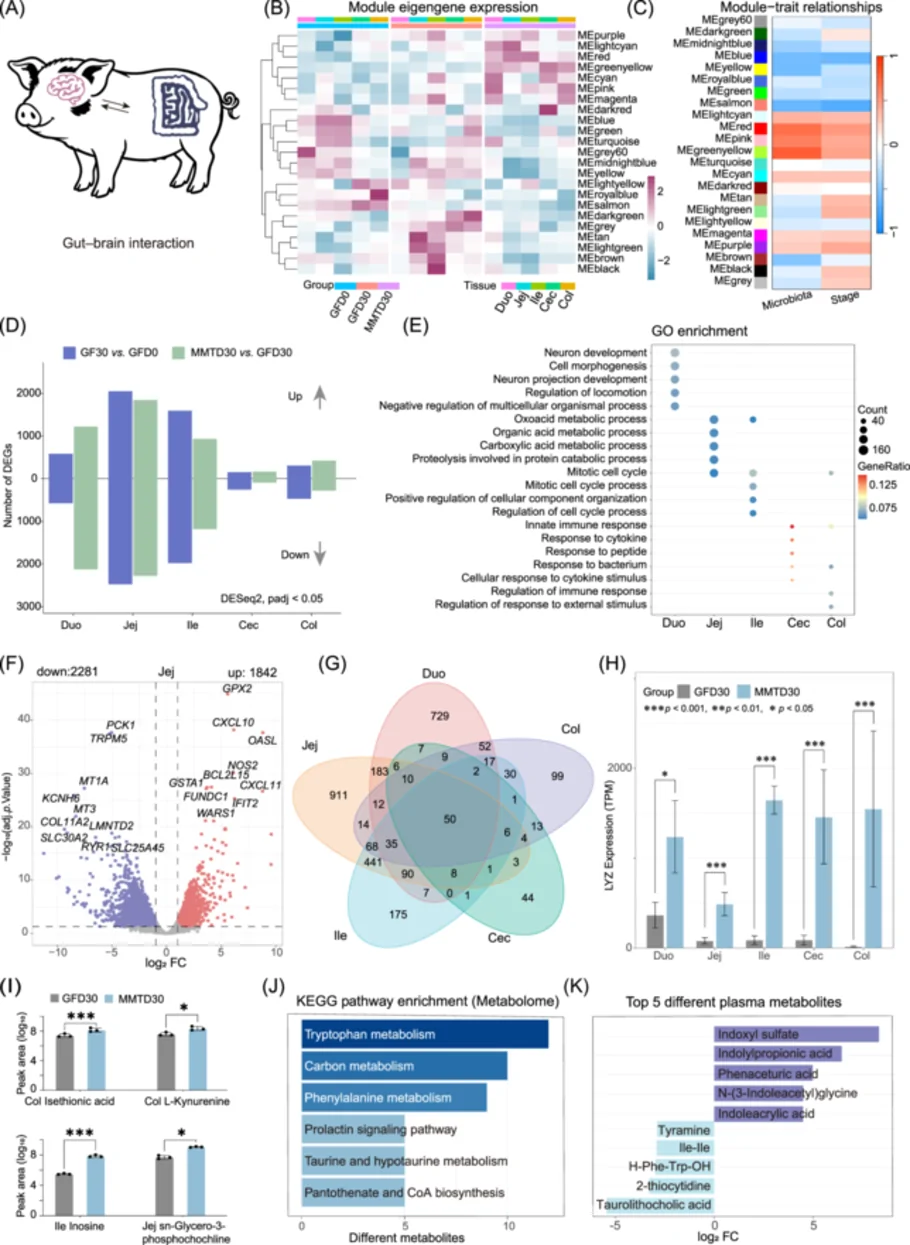
Maternal microbiota‐associated intestinal transcriptional and metabolic remodeling along the gut–brain axis. (A) Schematic representation of gut–brain interaction. (B) Heatmap showing WGCNA module eigengene expression across intestinal samples. Sample annotations indicate experimental group and intestinal segment. (C) Module–trait relationships between WGCNA modules and microbial status or developmental stage. Color indicates the correlation coefficient. (D) Numbers of upregulated and downregulated DEGs across the duodenum, jejunum, ileum, cecum, and colon under the MMTD30 versus GFD30 and GFD30 versus GFD0 comparisons. (E) GO enrichment of DEGs across the five intestinal segments in MMTD30 versus GFD30. The top five terms by GeneRatio are shown for each segment; dot size indicates gene count and color indicates GeneRatio. (F) Volcano plot of DEGs in the jejunum under the MMTD30 versus GFD30 comparison. Red and blue indicate upregulated and downregulated genes, respectively. (G) Five‐set Venn diagram showing 50 genes consistently upregulated across all five intestinal segments in the MMTD30 versus GFD30. (H) *LYZ* expression levels are shown as TPM (mean ± SEM). Statistical significance was determined by DESeq2 based on raw read counts, and P‑values were adjusted by Benjamini–Hochberg method. *p < 0.05, **p < 0.01, ***p < 0.001 (ns, not significant). (I) Relative abundances of representative differential metabolites in the jejunum, ileum, and colon of GFD30 and MMTD30 piglets. Metabolite abundance is shown as log_10_‐transformed peak area. Individual points represent biological replicates. *p < 0.05 and ***p < 0.001. (J) KEGG pathway enrichment of differential plasma metabolites between MMTD30 and GFD30. Bar length indicates the number of differential metabolites assigned to each pathway. (K) Top five increased and top five decreased plasma metabolites in MMTD30 versus GFD30. Positive log2FC values indicate higher abundance in MMTD30, whereas negative values indicate higher abundance in GFD30. Intestinal transcriptomic and metabolomic analyses were based on three independent piglets per experimental group (*n* = 3).

Differential expression analysis revealed pronounced segment‐specific transcriptional responses in both the MMTD30 versus GFD30 and GFD30 versus GFD0 comparisons, with substantially larger numbers of DEGs in the small intestine than in the cecum and colon (Figure 5D), indicating that the small intestine represents a major transcriptional interface responding to maternal microbiota exposure. For the MMTD30 versus GFD30 comparison, Gene Ontology (GO) enrichment further revealed distinct functional patterns across intestinal segments. Duodenal DEGs were primarily associated with cell morphogenesis and neuronal development‐related processes, jejunal DEGs were enriched in oxoacid, organic acid, and carboxylic acid metabolic processes, ileal DEGs were associated with cell‐cycle regulation, whereas cecal and colonic DEGs were predominantly enriched in immune‐, cytokine‐, bacterial‐response‐, and external‐stimulus‐related pathways (Figure 5E). Among all intestinal segments, the jejunum exhibited the most extensive transcriptional remodeling, with 1842 upregulated and 2281 downregulated genes in MMTD30 compared with GFD30 piglets (Figure 5F; Table S6). The corresponding volcano plots for the duodenum, ileum, cecum, and colon further illustrated marked differences in the magnitude and direction of transcriptional responses among intestinal segments (Figure S15B).

Cross‐segment comparison of the MMTD30 versus GFD30 response identified 50 genes that were consistently upregulated across all five intestinal segments (Figure 5G), whereas only seven genes were consistently downregulated (Figure S15C). STRING‐based analysis of the 50 shared upregulated genes revealed an interconnected network containing multiple interferon‐ and immune‐response‐associated genes, including *STAT1*, *ISG15*, *MX1*, *MX2*, *IFI44*, *USP18*, *OASL*, and IFIT‐family genes (Figure S15D). Among these conserved response genes, *LYZ* exhibited consistently elevated expression across all five intestinal segments in the MMTD30 piglets (Figure 5H), highlighting LYZ as a conserved marker of microbiota‐associated antimicrobial and innate immune remodeling.

To further characterize postnatal developmental changes under GF conditions, we examined the GFD30 versus GFD0 comparison. GO enrichment demonstrated marked segment specificity, with metabolic and ion‐transport processes represented in the duodenum and colon, neuron development and cell morphogenesis‐related processes in the jejunum and ileum, and cell‐motility‐ and G protein‐coupled receptor signaling‐related processes in the cecum (Figure S15E). Additional cross‐segment analysis identified six genes that were consistently upregulated and eight genes that were consistently downregulated across all five intestinal segments in GFD30 versus GFD0 (Figure S16A). Segment‐resolved differential‐expression analyses revealed pronounced heterogeneity in developmental transcriptional remodeling across intestinal segments (Figure S16B). GO enrichment analysis further identified distinct biological programs associated with individual intestinal regions, including neuron development in the jejunum, and ileum, and the G protein‐coupled receptor signaling pathway in the cecum (Figure S16C).

To determine whether systemic metabolic alterations were accompanied by local intestinal changes, we further analyzed metabolomic profiles of jejunum, ileum, cecum, and colon. Representative differential metabolites included isethionic acid and L‐kynurenine in the colon, inosine in the ileum, and sn‐glycero‐3‐phosphocholine in the jejunum (Figure 5I). These results demonstrate pronounced regional metabolic remodeling within the intestine following maternal microbiota transplantation. Plasma metabolomic analysis further revealed enrichment of differential metabolites in tryptophan metabolism, carbon metabolism, phenylalanine metabolism, prolactin signaling, taurine and hypotaurine metabolism, and pantothenate and CoA biosynthesis (Figure 5J). The top five increased (including indoxyl sulfate, indolylpropionic acid, phenaceturic acid, n‐(3‐indoleacetyl) glycine, and indoleacrylic acid) and decreased (including tyramine, Ile‐Ile, H‐Phe‐Trp‐OH, 2‐thiocytidine, and taurolithocholic acid) plasma metabolites in MMTD30 versus GFD30 piglets further demonstrated marked microbiota‐associated metabolic differences (Figure 5K). Collectively, these findings reveal that maternal microbiota transplantation establishes regional intestinal transcriptional and metabolic programs characterized by conserved antimicrobial signatures and altered tryptophan‐derived metabolite landscapes, providing molecular substrates potentially linking maternal microbiota exposure with coordinated gut–brain–neuroendocrine developmental programming.

### Cross‐species genetic enrichment reveals human trait‐associated signatures in microbiota‐responsive porcine cell populations

To assess the cross‐species relevance of the porcine multi‐organ cellular atlas, we integrated porcine cell‐type‐specific transcriptional signatures with human genome‐wide association study (GWAS) summary statistics using stratified linkage disequilibrium score regression (S‐LDSC) [31]. We generated 59 microbiota‐responsive cell‐type‐specific gene signatures derived from differentially expressed genes (DEGs) identified between MMTD30 and GFD30 across the AMY, HIP, HYP, BS, mPFC, PIT, and ADR. S‐LDSC revealed heterogeneous enrichment of human complex‐trait genetic signals across porcine neuronal, glial, vascular, endocrine, and immune cell populations (Figure 6A; Table S7). The analyzed traits included neuropsychiatric disorders (bipolar disorder, schizophrenia, ASD, and depression), cognitive traits (intelligence), neuroanatomical traits (AMY volume), sleep‐related traits (sleep duration), and neurodegenerative disorders (Alzheimer's disease), with significant enrichments displaying marked tissue‐ and cell‐type‐specific patterns.

**FIGURE 6:**
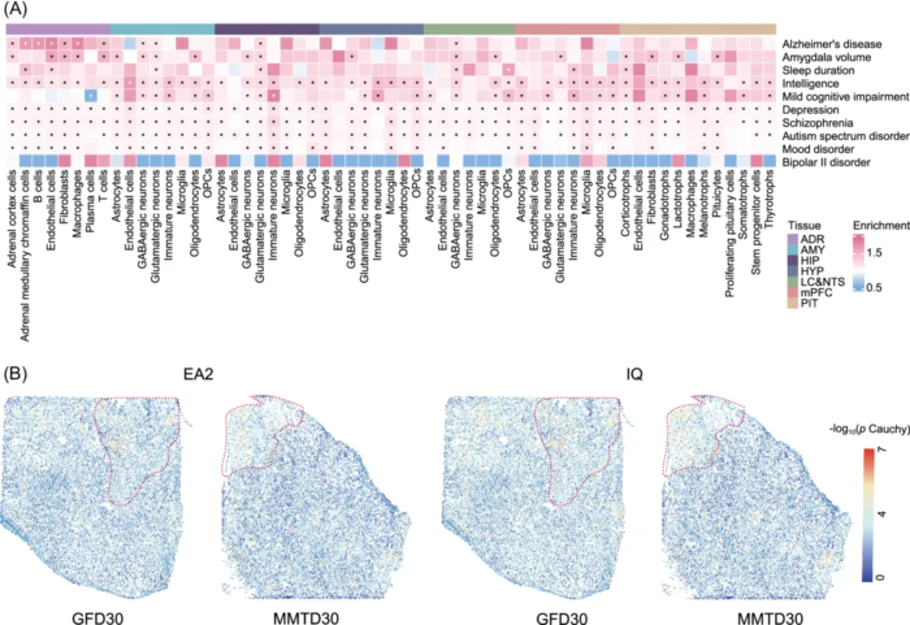
Cross‐species genetic enrichment and spatial distribution of human complex‐trait‐associated signals across the porcine brain and HPA axis. (A) Circular heatmap showing S‐LDSC enrichment of human GWAS‐derived genetic signals across tissue‐resolved cell populations from the AMY, HIP, HYP, BS, mPFC, PIT, and ADR. The analyzed traits include Bipolar II disorder, mood disorder, autism spectrum disorder, schizophrenia, mild cognitive impairment, depression, intelligence, sleep duration, amygdala volume, and Alzheimer's disease. Colors indicate per‐SNP enrichment estimates, and the outer annotation ring indicates the tissue of origin of each cell population. Asterisks indicate significant enrichment after Benjamini–Hochberg correction (FDR < 0.05). (B) Spatial visualization of Educational attainment (EA2)‐ and intelligence quotient (IQ)‐associated signals in GFD30 and MMTD30 spatial transcriptomic sections. Red dashed contours highlight the corresponding spatial regions. EA2, Educational attainment; FDR, false discovery rate; GFD30, germ‐free piglets at postnatal Day 30; GWAS, genome‐wide association study; IQ, intelligence quotient; MMTD30, maternal microbiota‐transplanted piglets at postnatal Day 30; S‐LDSC, stratified linkage disequilibrium score regression. Porcine cell‐type transcriptional annotations used for S‐LDSC were derived from three independent MMTD30 and three independent GFD30 piglets (*n* = 3 per group).

To further resolve trait‐associated patterns at a finer cluster level, we employed genetically informed spatial mapping of cells for complex traits (gsMap) [32] to spatial transcriptomic datasets generated from GFD30 and MMTD30 HYP sections. Trait‐associated enrichment patterns exhibited marked cluster‐ and group‐dependent distributions across the HYP (Figure S17A). Notably, educational attainment (EA2)‐ and intelligence quotient (IQ)‐associated signals showed prominent cluster‐level enrichment and spatial localization within HYP sections (Figure 6B). These findings indicate that human cognitive‐trait‐associated genetic architecture is preferentially represented within specific porcine cellular domains. Collectively, these cross‐species analyses demonstrate that microbiota‐responsive porcine brain and neuroendocrine cell populations capture genetic architectures associated with human cognitive, neuropsychiatric, neuroanatomical, sleep‐related, and neurodegenerative traits. These findings establish the pig as a translational large‐animal framework for dissecting conserved cellular mechanisms linking early‐life microbial exposure to brain‐related phenotypes.

## DISCUSSION

Our study explored how maternal microbiota transplantation is associated with remodeling of the developing porcine brain and neuroendocrine system. By employing a GF piglet model, our findings demonstrate that maternal microbiota exposure is associated with coordinated alterations in neuronal states, glial heterogeneity, neuron–glia communication networks, HPA‐axis regulation, and intestinal molecular programs.

This atlas revealed that neurons represented one of the major transcriptional targets associated with maternal microbiota exposure. In particular, neuronal populations from MMTD30 piglets displayed transcriptional characteristics associated with more differentiated glutamatergic states. Rather than indicating accelerated neuronal maturation, these findings suggest that maternal microbiota contributes to shaping developmental trajectories during early postnatal brain development. The observed neuronal remodeling was accompanied by extensive changes in inferred neuron–glia communication networks. CellChat analysis suggested microbiota‐associated modulation of enhanced NRG1/3–ERBB4 interactions in MMTD30 piglets, accompanied by altered glutamate–glutamine metabolic profiles. Previous studies have demonstrated that NRG1 activates nNOS via ErbB4 to regulate GABA release [33], and microbial colonization contributes to neuronal maturation and neurotransmitter‐related metabolic regulation [23, 29], supporting a potential link between microbiota‐associated signaling and neural circuit development [34, 35]. However, the precise microbial taxa or metabolites responsible for these effects remain to be determined.

Beyond neurons, our study highlights the substantial plasticity of glial populations during early development [36, 37]. Gut‐derived microbial metabolites have also been shown to influence oligodendrocyte‐related programs and brain function, further supporting the sensitivity of multiple glial lineages to microbial signals [38]. Astrocytes, microglia, and oligodendrocytes displayed distinct region‐specific transcriptional responses and regulatory architectures following maternal microbiota transplantation. In particular, astrocytes exhibited marked heterogeneity, with six transcriptional states associated with metabolic regulation, synaptic modulation, cAMP/PKA signaling, and developmental programs. Recent single‐cell studies have similarly demonstrated that developing astrocytes occupy heterogeneous transcriptional states and that their specification is shaped by both developmental origin and local environmental cues [39]. These findings extend previous observations that the microbiota shapes microglial maturation and that microbiota‐derived metabolites can regulate microglia–astrocyte interactions, supporting the broader involvement of multiple glial lineages in microbiota‐associated remodeling [40, 41].

The HPA axis represents another important interface linking microbial signals, immune regulation, and brain development [42]. Across the HYP, PIT, and ADR, maternal microbiota transplantation was associated with coordinated changes in cellular communication networks and shared stress‐response transcriptional programs. The reduced plasma cortisol levels further support microbiota‐associated modulation of neuroendocrine homeostasis and stress response [42, 43]. However, because behavioral assays were not included in the present study, we cannot determine whether reduced cortisol concentrations correspond to measurable alterations in stress‐related behaviors. Thus, our results indicate altered HPA‐axis activity rather than direct evidence of behavioral adaptation. This concept aligns with previous evidence that gut‐derived microbial metabolites can mimic host signaling molecules to override host sensory decisions, and thereby promotes fitness of both the host and the microbiota [44].

Early‐life microbial colonization profoundly influences immune maturation [45], and energy homeostasis can communicate with the central nervous system through cytokines, immune cells, tryptophan metabolism, and neuroendocrine pathways. An important challenge is therefore to distinguish direct microbial‐derived signals from indirect effects mediated through immune maturation [46]. Our additional cytokine profiling provides evidence that maternal microbiota transplantation induced coordinated peripheral immune remodeling. MMTD30 piglets exhibited increased circulating IL‐6, and TNF‐α, together with regulatory cytokines including IL‐10 and IL‐1ra, consistent with reported differences in immune regulation between GF and SPF piglets [47]. This pattern does not indicate a simple inflammatory activation state but instead suggests a dynamic immune maturation process involving both immune remodeling and regulatory adaptation. Therefore, the observed neural and endocrine phenotypes likely reflect the combined influence of microbial metabolites, immune maturation, intestinal responses, and neuroendocrine communication rather than a single dominant pathway.

The intestine represents a major interface through which maternal microbiota may influence host physiology. Our intestinal transcriptomic analysis revealed strong segment‐specific responses following microbiota transplantation, with the jejunum displaying the largest transcriptional remodeling. This pattern likely reflects the distinct physiological functions of different intestinal regions, rather than simply differences in responsiveness. The stronger transcriptional response observed in the small intestine may reflect its specialized role as an absorptive, nutrient‐sensing, immune, and neuroepithelial interface [48]. Through interactions involving vagal signaling, enteroendocrine pathways, microbial metabolites (e.g., indole), and immune mediators, small‐intestinal remodeling may provide multiple routes for energy metabolism between the gut and developing brain [49]. Notably, maternal microbiota‐induced metabolic remodeling was most pronounced in the jejunum and ileum, consistent with previous studies identifying the small intestine as a key interface for gut–brain communication [48].

Metabolomic analyses further identified alterations in tryptophan‐related metabolites, including indoxyl sulfate and indolylpropionic acid, following maternal microbiota transplantation. Tryptophan‐derived microbial metabolites, including indole derivatives, can engage AHR‐dependent pathways in both intestinal and central nervous system cell populations, including astrocytes [50, 51]. Together with altered glutamate–glutamine metabolism, these findings suggest that microbiota‐associated metabolic remodeling may contribute to neurodevelopment through multiple interconnected biochemical pathways and/or vagal signaling. However, our data do not establish direct causal relationships between individual metabolites and specific brain phenotypes.

A major advantage of the porcine model is its anatomical, physiological, and developmental similarity to humans compared with conventional rodent models [5, 52, 53]. To evaluate the translational relevance of our cellular atlas, we integrated porcine cell‐type‐specific transcriptional signatures with human GWAS datasets using S‐LDSC. The analysis revealed enrichment of human complex‐trait‐associated genetic signals across specific porcine neuronal, glial, vascular, endocrine, and immune populations. These associations were observed for multiple neuropsychiatric, cognitive [54], neuroanatomical, and sleep‐related traits, suggesting evolutionary conservation of cellular programs underlying human brain‐related phenotypes. Importantly, these genetic enrichments should not be interpreted as indicating that maternal microbiota increases or decreases disease susceptibility. Instead, they reflect the degree to which porcine cell‐type transcriptional programs overlap with human trait‐associated genetic architectures.

Several limitations should be acknowledged. The biological sample size was limited due to the technical challenges of maintaining GF large‐animal models, with three independent animals per group used as biological replicates for all analyses. In addition, future spatial profiling across additional brain regions and developmental stages will further elucidate the spatiotemporal dynamics of microbiota‐mediated neurodevelopment. Also, although our multi‐omics approach reveals coordinated remodeling across the gut–brain–endocrine axis, most regulatory relationships remain associative. Future studies incorporating larger cohorts, targeted microbial manipulation [55], metabolite intervention, immune perturbation, and behavioral assessments will be required to establish causal mechanisms. Nevertheless, this study provides a comprehensive cellular and molecular resource highlighting the role of maternal microbiota‐associated signals in early mammalian neurodevelopment and establishes the pig as a valuable translational model for dissecting conserved mechanisms linking microbiota, brain function, and human complex traits.

## CONCLUSIONS

In summary, we generated a multi‐regional single‐nucleus and spatial transcriptomic atlas to characterize maternal microbiota‐associated remodeling of the developing porcine brain. Our integrated analyses revealed that maternal microbiota transplantation is associated with coordinated changes in neuronal maturation, neuron–glia communication, glial regulatory programs, and HPA‐axis stress‐related transcriptional responses. In parallel, intestinal transcriptional and metabolic analyses demonstrated segment‐specific remodeling along the microbiota–gut–brain axis. Cross‐species genetic enrichment further linked porcine brain cell populations to human neuropsychiatric, cognitive, and neurological traits. Together, this study provides a cellular framework for understanding how early‐life maternal microbiota influences brain development and establishes the pig as a translational model for microbiota–brain interaction research.

## METHODS AND MATERIALS

### Ethical approval and experimental design

The experimental protocol was approved by the Chongqing Academy of Animal Sciences and the Institute of Subtropical Agriculture, Chinese Academy of Sciences (Approval No. XKY‐20240630) This study was conducted at the Chongqing Academy of Animal Sciences using a controlled GF piglet experimental system. A total of nine Rongchang piglets were included in the study. Three GF piglets were euthanized and sampled at postnatal Day 0 (GFD0) to establish the baseline developmental profile. The remaining six piglets were randomly allocated into two groups (*n* = 3 per group): a GF group maintained in sterile isolators until postnatal Day 30 (GFD30) and a maternal microbiota transplantation group receiving maternal microbiota transplantation and maintained until postnatal Day 30 (MMTD30). To prevent potential cross‐contamination between experimental conditions, GFD30 and MMTD30 piglets were housed separately in identical sterile isolators under continuous environmental monitoring and strict GF management procedures.

### Microbiota inoculation, sterility validation, and sample collection

A standardized maternal microbiota inoculum was prepared from fecal samples collected from a healthy donor sow. In this study, maternal microbiota transplantation refers to the experimental introduction of sow‐derived fecal microbiota into germ‐free piglets by oral gavage and is distinct from natural maternal microbial transmission. Beginning at postnatal Day 7, MMTD30 piglets received 1 mL of the fecal microbiota suspension per piglet per day by oral gavage for three consecutive days, whereas GFD30 piglets received an equivalent volume of sterile culture medium following the same schedule. All piglets were fed a vacuum‐sterilized diet and monitored daily for general health status. Samples from GFD30 piglets were subjected to microbiological culture to verify GF status, whereas samples from MMTD30 piglets were analyzed to confirm successful microbial colonization. All samples were transferred under sterile conditions and stored at −80°C until further analysis. Systemic tissue collection was performed at postnatal Days 0 and 30. Brain tissues, including the HYP, brainstem region containing the BS, AMY, HIP, and mPFC, were collected according to the stereotaxic atlas of the pig brain [56]. The PIT and ADR were also harvested. Intestinal tissues included the duodenum, jejunum, ileum, cecum, and colon. Plasma was obtained by centrifugation at 3000 × *g* for 15 min at 4°C and stored at −80°C until analysis. Serum was separated from clotted blood by centrifugation at 3000 × *g* for 15 min at 4°C and stored at −80°C until cytokine analysis. The experimental setup was illustrated in the figures with a cartoon pig image from BioRender.com.

### Nucleus preparation and snRNA‐seq library construction and sequencing

Tissues were homogenized in ice‐cold homogenization buffer containing IGEPAL CA‐630 (Sigma, I8896), MgCl2 (Macklin, M1028), NaCl (Solarbio, S8210), and RNase inhibitor (Sigma, 3335399001) using a glass Dounce homogenizer for 2 min on ice. Homogenates were filtered through a 40‐μm cell strainer and centrifuged at 500 × *g* for 5 min at 4°C. Nuclear pellets were resuspended in PBS containing RNase inhibitor. Nuclear concentration and integrity were evaluated using a SeekMate Titan Fluorescence Cell Counter (SeekGene, M002C) with AO/PI staining and by microscopic examination following trypan‐blue staining.

snRNA‐seq libraries were prepared using the SeekOne DD Single Cell 3' Library Preparation Kit (SeekGene, K00202). Nuclei were mixed with reverse‐transcription reagents and loaded onto SeekOne DD Chip S3 together with barcoded hydrogel beads and partitioning oil. Reverse transcription was performed at 42°C for 90 min, followed by inactivation at 85°C for 5 min. cDNA was recovered, amplified, purified, fragmented, end‐repaired, A‐tailed, adaptor‐ligated, and subjected to indexed PCR. Final libraries were purified using VAHTS DNA Clean Beads (Vazyme, N411‐01), evaluated using Qubit and a Bio‐Fragment Analyzer, and sequenced on the GeneMind SURFSeq 5000 platform with paired‐end 150 bp reads.

### snRNA‐seq data preprocessing, integration, and dimensionality reduction

Raw sequencing reads were aligned to the Sus scrofa reference genome (Sscrofa11.1) and quantified using SeekSoulTools (v1.2.1). Downstream analyses were performed in R (v4.4) using Seurat [57] (v5.2.0). Ambient RNA contamination was estimated using decontX in the celda package (v1.16.0), and cells with contamination fractions > 80% were removed. Doublets were identified using DoubletFinder (v2.0.3) with an expected doublet rate of 8%. Cells were retained when UMI and detected‐gene counts fell within the 5th–95th percentile range and mitochondrial transcripts accounted for ≤20% of total expression.

Quality‐controlled data were normalized using the LogNormalize method in Seurat, with total expression scaled to 10,000 transcripts per cell, followed by log transformation. The top 2000 highly variable genes were identified using FindVariableFeatures. Batch effects were corrected using Harmony. Principal component analysis (PCA) was performed on the integrated dataset, and the first 30 principal components were used for subsequent clustering and dimensionality reduction.

### Cell clustering, cell‐type annotation, and subcluster analysis

Cell clustering was performed using FindNeighbors and FindClusters in Seurat (v5.2.0), and UMAP was used for two‐dimensional visualization. Major cell types were annotated based on canonical marker genes together with cluster‐specific markers identified using FindAllMarkers (|avg_log2FC| > 0.25, min.pct ≥ 0.25, and Bonferroni‐adjusted *p* < 0.05). Marker expression patterns were visualized using feature plots, violin plots, and dot plots.

To resolve heterogeneity within selected major cell populations, target cell subsets were extracted using the subset function in Seurat and independently reprocessed for normalization, highly variable gene selection, PCA, neighborhood construction, and clustering. Subcluster‐specific marker genes were identified using the same FindAllMarkers thresholds, and subclusters were annotated according to established marker genes and relevant literature.

### Differential expression and functional enrichment analysis

DEGs between the MMTD30 and GFD30 groups were identified using the FindMarkers function in Seurat (v5.4.0) with a two‐sided Wilcoxon rank‐sum test, performed separately within each annotated cell type for each brain region (AMY, HIP, HYP, BS, and mPFC). Across all three experimental groups, 45 single‐nucleus libraries were generated in total; the MMTD30‐versus‐GFD30 comparison included 15 libraries per group. The Wilcoxon test evaluated nucleus‐level expression distributions within each cell‐type‐ and region‐specific comparison; individual nuclei served as the statistical unit and were not regarded as independent biological replicates. Genes detected in at least 25% of nuclei in either group (min.pct = 0.25) were tested, with logfc.threshold = 0.25. Genes with |avg_log2FC| ≥ 0.25 and Bonferroni‐adjusted *p* < 0.05 were considered significantly differentially expressed. Significant DEGs from each cell‐type‐ and tissue‐specific comparison were visualized as marker‐effect plots using the marker_effect_plot function in the scMarkerViz package (v0.1.1; 10.5281/zenodo.22010312). Significant DEGs were subsequently subjected to Gene Ontology (GO) Biological Process enrichment analysis using the clusterProfiler package with the porcine annotation database org.Ss.eg.db. Enrichment analysis was performed using Benjamini–Hochberg multiple‐testing correction (pAdjustMethod = “BH”, pvalueCutoff = 0.05, and qvalueCutoff = 0.2), and the top five enriched terms were retained for visualization for each cell type and tissue.

### Pseudotime trajectory analysis

Pseudotime trajectory analysis was performed using Monocle 3 [58] (v1.4.27). The integrated neuronal dataset was subset to glutamatergic neurons, GABAergic neurons, and immature neurons, and downsampled to 10,000 cells while preserving representation of each experimental group. A cell data set was constructed using log‐normalized expression, followed by PCA with 50 dimensions and UMAP embedding. Trajectories were learned using reversed graph embedding and the learn_graph function. Immature neurons were designated as the root population, and cells were ordered along pseudotime using order_cells. Astrocyte subclusters A1–A6 were additionally subjected to pseudotime trajectory analysis using Monocle 3 following the same trajectory‐inference framework described above. The A1 subcluster was designated as the root population, and cells were ordered along the inferred astrocyte pseudotime trajectory.

### Cell‐cell communication analysis

Cell–cell communication was inferred using CellChat [59] (v2.2.0) with the CellChatDB.human ligand–receptor database restricted to the “Secreted Signaling” subset. For each condition (GFD30 and MMTD30), snRNA‐seq data comprised nuclei derived from three independent piglets (biological replicates, *n* = 3 per group), which were pooled to construct one group‐level CellChat object per condition; accordingly, individual animals were not treated as independent statistical units, and all results describe condition‐level communication patterns rather than animal‐level statistical inference. Communication probabilities were computed with population‐size correction enabled (population.size = TRUE), overexpressed ligand–receptor genes were identified per cell type using CellChat's default Wilcoxon‐based thresholds, and interactions supported by fewer than 10 cells were removed with filterCommunication (min.cells = 10). The two group‐level objects were aligned to their common cell types and signaling pathways and merged with mergeCellChat, after which shared and condition‐specific ligand–receptor pairs were identified. Shared interactions were visualized as pathway‐colored probability scatter plots, condition‐specific interactions were summarized across source–target cell‐type combinations as divergent bar plots, and representative ligand–receptor pairs with the largest between‐condition probability differences were displayed in ComplexHeatmap panels with signaling‐pathway annotations. Cell–cell communication networks were visualized as circle plots in which edge width denotes the number of inferred interactions or the cumulative interaction score.

### Transcription factor regulon analysis

Transcription factor regulons were inferred using pySCENIC [60] (v0.12.1). For each tissue–group combination, expression matrices were downsampled to a maximum of 500 cells. Genes detected in fewer than three cells were excluded, and the top 5000 highly variable genes were retained. Porcine genes were converted to human one‐to‐one orthologs using Ensembl BioMart to enable the use of human cisTarget databases. GRNBoost2 was used for co‐expression network inference, followed by cisTarget motif enrichment using the hg38 v10 ranking databases covering 500‐bp and 10‐kb upstream regulatory regions with dropout masking enabled. Regulon activity was quantified using AUCell.

Regulon specificity was evaluated using difference‐based and Jensen–Shannon divergence‐based regulon specificity scores. A regulon was assigned to a cell type when its normalized cell‐type contribution exceeded 0.5. Regulons were grouped into co‐activity modules using Ward.D2 hierarchical clustering, with the optimal module number selected according to the silhouette criterion. GO enrichment of module target genes was performed using clusterProfiler (v4.14.6) with org.Ss.eg.db, and Benjamini–Hochberg‐adjusted *p* < 0.05 was considered statistically significant.

### *In silico* knockout analysis

Virtual gene knockout analyses were performed in astrocytes using the scTenifoldKnk. For each target gene, 1000 cells were randomly sampled, and the top 1000 highly variable genes were selected using the variance‐stabilizing transformation implemented in Seurat (v5). Tensor‐decomposition‐based gene regulatory networks were constructed using 30 random network realizations (nc_nNet = 30), and edges were sparsified at quantile *q* = 0.99. Differentially regulated genes following virtual knockout were identified using *p* < 0.05 and regulatory distance > 1 × 10^−10^. GO enrichment was performed using clusterProfiler (v4.14.6) with org.Ss.eg.db, and Benjamini–Hochberg‐adjusted *p* < 0.05 was considered significant.

### Correlation and cell enrichment analyses

Average expression profiles of major cell types were calculated using AverageExpression in Seurat (v5.2.0). Pairwise Pearson correlation coefficients were calculated from the resulting cell type × gene expression matrix using the cor function in R (v4.4), and the correlation matrix was visualized using pheatmap (v1.0.13).

Cell‐distribution preferences across experimental conditions were further evaluated using the observed‐to‐expected ratio (Ro/e). For each cell population, the observed number of cells was compared with the number expected according to the overall cell distribution. Statistical enrichment was assessed using a hypergeometric test, and *p* values were adjusted using the Benjamini–Hochberg procedure. Adjusted *p* < 0.05 was considered statistically significant.

### Cross‐species genetic enrichment analysis using S‐LDSC

Cell‐type‐specific gene sets were derived from porcine single‐nucleus RNA‐sequencing (snRNA‐seq) data by retaining DEGs between MMTD30 and GFD30 with Bonferroni‐adjusted *p* < 0.05 (up‐ and down‐regulated DEGs combined), yielding 59 region‐resolved gene sets across the ADR, AMY, HIP, HYP, BS, mPFC, and PIT (54–4752 genes per set). The porcine snRNA‐seq data were obtained from three independent MMTD30 and three independent GFD30 piglets (*n* = 3 biological replicates per group); individual nuclei were not treated as independent biological replicates. Porcine gene symbols were matched to human gene symbols, and human gene coordinates were assigned based on GRCh37 (genes without a matching human gene symbol were excluded). GWAS summary statistics for 10 human traits were obtained from the GWAS Catalog, and variants within the extended MHC region (chr6:25–35 Mb, GRCh37) were excluded. For each gene set, SNPs located within ±100 kb of annotated genes were used to construct binary annotations (overlapping intervals merged). LD scores were calculated using the 1000 Genomes Project Phase 3 European reference panel (*n* = 489), restricted to HapMap3 SNPs with a 1‐cM LD window. S‐LDSC [31] was performed using ldsc.py (v1.0.0) with the 97‐annotation baseline‐LD v2.2 model plus the cell‐type annotation (98 categories in total), ‐‐overlap‐annot, and 200 jackknife blocks. Enrichment significance was assessed using Benjamini–Hochberg correction, with FDR < 0.05 considered significant. Statistical inference in S‐LDSC was based on GWAS summary statistics and block jackknife estimation rather than on the number of porcine nuclei.

### Stereo‐seq tissue preparation, library construction, and sequencing

Fresh hypothalamic tissues were immediately embedded in OCT compound and snap‐frozen on dry ice or in liquid nitrogen to preserve tissue morphology and RNA integrity. OCT‐embedded tissues were equilibrated in a cryostat and sectioned at a thickness of 10 μm before placement onto the 0.5 cm × 0.5 cm capture area of Stereo‐seq chips. Pre‐permeabilization testing was performed before formal experiments, and the optimal permeabilization time was determined to be 18 min.

Sections were fixed with precooled methanol at −20°C for 30 min and stained with ssDNA reagent for 5 min at room temperature. High‐resolution fluorescence images were acquired before permeabilization. Sections were treated with permeabilization solution for 18 min at 37°C, allowing released mRNA molecules to hybridize to spatially barcoded capture probes containing coordinate identifiers, molecular identifiers, and poly(T) sequences. Reverse transcription was performed at 45°C for 2 h, followed by cDNA release, neutralization, PCR amplification, bead purification, and library construction. Libraries were sequenced on the DNBSEQ platform.

### Stereo‐seq data processing and cell‐type annotation

Stereo‐seq data were processed using the SAW pipeline. Spatial coordinate identifiers and molecular identifiers in Read 1 were extracted, while cDNA reads were aligned to the Sscrofa11.1 reference genome and quantified to generate spatial gene‐expression matrices. ssDNA images were registered with sequencing coordinates, and CellBin was used for cell segmentation and extraction of cell‐level gene‐expression profiles. The resulting cell‐level expression matrix was converted to cellbin.gem format for downstream analysis.

Cell‐level expression matrices were converted using StereoPy and SeuratDisk for analysis in Seurat (v5.2.0). Expression data within manually annotated regions of interest were extracted and normalized using SCTransform. Cell‐type annotation was performed by label transfer using the quality‐controlled snRNA‐seq dataset as the reference. FindTransferAnchors and TransferData were used to transfer cell‐type labels to spatially resolved cells, enabling visualization of the in situ distribution of major cell populations in the HYP.

### Spatial mapping of human trait‐associated signals using gsMap

Spatial transcriptomic data from porcine HYP sections were analyzed using gsMap to identify spatial regions associated with human complex traits [32]. Raw count matrices were normalized and standardized, and highly variable genes were selected for analysis. Porcine genes were converted to human orthologs using biomaRt. gsMap integrated gene expression profiles and spatial coordinates using a graph‐based attention autoencoder to identify spatially coherent transcriptional domains and calculate gene specificity scores. Human GWAS summary statistics were used to evaluate trait‐associated genetic enrichment across spatial domains. SNP annotations were assigned based on genomic proximity and regulatory links, and spatial heritability enrichment was assessed using S‐LDSC with the 1000 Genomes Phase 3 LD reference panel. For cluster‐level analyses, association *p* values of individual spatial spots within each cluster were aggregated using the Cauchy combination test to obtain cluster‐level *P* Cauchy values.

### Intestinal transcriptome sequencing and differential expression analysis

Total RNA was extracted from intestinal tissues using TRIzol Reagent (Invitrogen) according to the manufacturer's instructions. RNA concentration was measured using a Qubit 3.0 fluorometer (Life Technologies) and a Qubit RNA Broad Range Assay Kit. Stranded RNA‐seq libraries were constructed using 2 μg of polyadenylated RNA per sample and sequenced on an Illumina NovaSeq. 6000 platform using paired‐end 150‐bp sequencing.

Raw sequencing reads were quality‐filtered and aligned to the Sscrofa11.1 reference genome using HISAT2 [61] (v2.2.1), followed by processing with SAMtools [62] (v1.22). Gene expression levels were quantified using FeatureCounts [63] (v2.1.1), and PCA was performed to evaluate transcriptomic variation among samples. Differential expression analysis was performed using DESeq2 [64] (v1.46.0), with DEGs defined by |log2FC| ≥ 1 and FDR ≤ 0.05. Functional enrichment analysis was performed using clusterProfiler (v4.10.0).

### Weighted gene co‐expression and protein–protein interaction network analyses

Weighted gene co‐expression network analysis (WGCNA) was performed using intestinal RNA‐seq expression profiles to identify coordinated transcriptional modules across intestinal segments and experimental conditions [65]. A signed gene co‐expression network was constructed using a soft‐thresholding power selected according to the scale‐free topology criterion. The adjacency matrix was transformed into a topological overlap matrix, and gene modules were identified by hierarchical clustering and dynamic tree cutting. Modules with similar expression profiles were subsequently merged according to eigengene similarity. Module eigengenes, defined as the first principal component of each module, were calculated to summarize module‐level expression patterns. Module–trait relationships were evaluated by correlating module eigengenes with microbial status and developmental stage, and corresponding correlation coefficients and *p* values were visualized using module‐trait heatmaps.

### Protein–protein interaction

The 50 genes consistently upregulated across all five intestinal segments in MMTD30 compared with GFD30 piglets were further subjected to protein–protein interaction analysis using the STRING database. The resulting interaction network was used to characterize functional connectivity among the shared upregulated genes.

### Metabolomics analysis

Plasma metabolomic profiling was performed using an ExionLC™ AD UPLC system coupled to a QTRAP® 6500+ mass spectrometer (SCIEX). Plasma metabolites were extracted with acetonitrile–methanol solution and analyzed by LC–MS/MS in positive‐ and negative‐ion modes. Metabolites were identified against a standard compound reference database and quantified in multiple reaction monitoring (MRM) mode.

Untargeted metabolomic profiling of intestinal mucosal samples was performed by Novogene Co. Ltd. using LC–MS/MS. Pooled quality‐control samples were prepared by mixing equal volumes of samples and were inserted throughout the analytical sequence to monitor system stability. Raw MS data were converted to mzXML format using ProteoWizard and processed using XCMS for peak detection, alignment, and retention‐time correction. Peaks with missing values in >50% of samples within any group were removed, and metabolites were annotated against the Novogene in‐house database.

For differential analysis, multivariate analyses including PCA and OPLS‐DA were performed. Differential metabolites were identified based on variable importance in the projection (VIP) > 1.0, fold change (FC) > 1.2 or FC < 0.833, and *p* < 0.05, with *p* values calculated using Student's t‐test. Pathway enrichment analysis of differential metabolites was performed using the KEGG database.

### Biochemical assays

Plasma glutamic acid and glutamine were analyzed by Metware Biotechnology using ultra‐performance liquid chromatography‐tandem mass spectrometry. After extraction with methanol/acetonitrile/water and protein precipitation at −20°C, supernatants were collected by centrifugation at 12,000 rpm for 15 min. Mass spectrometric detection was performed in MRM mode. Data were processed using Analyst software, and absolute concentrations were calculated using internal standard‐normalized calibration.

Plasma cortisol concentrations were determined using a competitive enzyme‐linked immunosorbent assay (ELISA) kit (H094‐1‐1; Nanjing Jiancheng Bioengineering Institute) according to the manufacturer's instructions.

Serum cytokine concentrations were measured using a porcine multiplex cytokine assay kit (UNIV) on a Luminex® platform according to the manufacturer's instructions. Serum samples were loaded at 60 μL per well, and each biological sample was measured in three technical replicates. The cytokines included IL‐6, IL‐1ra, IL‐10, and TNF‐α, and their concentrations were calculated from the corresponding standard curves.

### Immunofluorescence staining and image analysis

Immunofluorescence staining was performed on frozen sections to detect the distribution and expression of target proteins in brain tissues. Briefly, brain tissues were fixed in 4% PFA and cryoprotected in 30% sucrose‐PBS solution, followed by cryosectioning into 40 μm‐thick sections. The tissue sections were incubated overnight at 4°C with primary antibodies, including rabbit anti‐IBA‐1 (1:500, Ab178847, Abcam), rabbit anti‐VGAT (1:500, Ab308062, Abcam). After thorough washing with PBS, sections were incubated with corresponding secondary antibodies, including Alexa Fluor Plus 488‐conjugated secondary antibody (1:200, A32731, Thermo Fisher Scientific) for 2 h at room temperature. Nuclei were counterstained with 4',6‐diamidino‐2‐phenylindole (DAPI, 0.4 μg/mL, Sigma) for 10 min. All sections were mounted with Fluoromount™ Aqueous Mounting Medium (F4680, Sigma). Fluorescence images were acquired using an Olympus VS120 virtual slide scanning system with consistent excitation and emission parameters for DAPI (360–370 nm excitation/420–460 nm emission), green fluorescence (460–495 nm excitation/510–550 nm emission). For quantitative analysis of GABAergic neuron populations, cell counting was carried out manually. The relative fluorescence intensity and positive area percentage were quantitatively analyzed using ImageJ software.

### Statistical analysis

Statistical analyses were performed using R unless otherwise specified. Unless otherwise specified, biological replication was defined at the animal level, with three independent piglets per experimental group (*n* = 3). Individual nuclei or cells were not considered independent biological replicates. For cell‐level computational analyses, nuclei or cells served as analytical observations within the corresponding algorithms, whereas the biological sample size remained *n* = 3 piglets per group. snRNA‐seq differential expression between experimental groups was performed using the FindMarkers function in Seurat with the Wilcoxon rank‐sum test as described above, with Bonferroni‐adjusted *p* < 0.05 considered statistically significant. Multiple‐testing correction was performed using the Benjamini–Hochberg method where applicable, with FDR < 0.05 considered statistically significant. Plasma glutamic acid, glutamine, and cortisol concentrations, as well as serum cytokine concentrations, were compared between GFD30 and MMTD30 piglets using two‐sided Student's *t*‐tests. For metabolomic comparisons, OPLS‐DA was used to calculate VIP scores, and Student's *t*‐tests were used to calculate *p* values; differential metabolites were defined using the criteria described above. Statistical procedures specific to individual bioinformatic analyses are described in the respective Methods sections.

## AUTHOR CONTRIBUTIONS

**Leli Wang**: Conceptualization; investigation; funding acquisition; writing—original draft; writing—review and editing; resources; supervision; formal analysis; project administration; validation; visualization; methodology; software. **Chao Yan**: Data curation; visualization; writing—original draft. **Lei Li**: Validation; writing—original draft; data curation; resources; writing—review and editing. **Yuankun Deng**: Writing—review and editing. **Hua Shao**: Investigation. **Kuo Long**: Investigation; resources; visualization; validation; writing—original draft. **Cimin Long**: Investigation. **Yuanyuan Zhu**: Writing—review and editing; writing—original draft; visualization; resources. **Meiqing Chen**: Visualization. **Jialu Huang**: Investigation; visualization; resources; validation; writing—original draft. **Jinghua Jiang**: Investigation; validation; visualization. **Ziyi Lin**: Investigation; validation. **Yunying Yao**: Software; writing—review and editing. **Tao Yang**: Software; writing—review and editing. **Wenbin Guo**: Software; writing—review and editing; visualization; supervision. **Liangpeng Ge**: Investigation; resources. **Jing Sun**: Investigation; validation. **Yuchun Ding**: Investigation; validation. **Zuohua Liu**: Investigation; resources. **Mingzhu Yin**: Investigation; resources. **Yulong Yin**: Investigation; supervision.

## CONFLICT OF INTEREST STATEMENT

The authors declare no conflicts of interest.

## ETHICS STATEMENT

All animal procedures were approved by the Animal Ethics Committee of Chongqing Academy of Animal Science and the Institute of Subtropical Agriculture, Chinese Academy of Sciences (Approval No. XKY‐20240630).

## Supporting information


**Figure S1:** Regional distribution and functional characterization of the six major brain cell populations.
**Figure S2:** Spatial transcriptomic characterization of major cell populations in the HYP.
**Figure S3:** Neuronal subtype markers, composition, developmental trajectories, and regional distribution.
**Figure S4:** Neuronal subtype‐specific transcriptional responses across brain regions and experimental conditions.
**Figure S5:** Immunofluorescence characterization and quantification of *VGAT*‐positive GABAergic cells in the HYP.
**Figure S6:** Region‐specific remodeling of inferred neural–glial communication between GFD30 and MMTD30 piglets.
**Figure S7:** Distribution, proportion changes, functional annotation, and region‐specific transcriptional responses of major glial populations.
**Figure S8:** Developmental‐stage‐associated differential expression in major glial populations across brain regions.
**Figure S9:** Immunofluorescence characterization of *IBA1*‐positive microglia in the HYP.
**Figure S10:** Transcriptional heterogeneity and inferred developmental trajectories of astrocyte subclusters.
**Figure S11:** Region‐ and subtype‐specific astrocyte transcriptional responses associated with maternal microbiota transplantation.
**Figure S12:** In silico knockout analyses of *FOXO1* and *LMO1* in MMTD30 astrocytes.
**Figure S13:** Cell‐type composition and inferred intercellular communication across the HPA axis.
**Figure S14:** Cell‐type‐resolved transcriptional responses and HPA‐related gene expression across the HPA axis.
**Figure S15:** Intestinal transcriptional variation, shared differential‐expression patterns, and functional enrichment across developmental and microbial conditions.
**Figure S16:** Intestinal transcriptional responses across developmental and microbial conditions.
**Figure S17:** gsMap‐based analysis of major‐cell‐lineage and cluster‐level associations with human complex traits in the porcine brain.

**Table S1:** Marker genes used for cell‐type annotation across the porcine brain and HPA‐axis snRNA‐seq datasets.
**Table S2:** Differentially expressed genes across tissues and cell populations in the MMTD30 versus GFD30 comparison.
**Table S3:** Gene Ontology Biological Process enrichment of differentially expressed genes in the MMTD30 versus GFD30 comparison.
**Table S4:** Differentially expressed genes across tissues and cell populations in the GFD30 versus GFD0 comparison.
**Table S5:** Gene Ontology Biological Process enrichment of differentially expressed genes in the GFD30 versus GFD0 comparison.
**Table S6:** Differentially expressed genes across five intestinal segments in the MMTD30 versus GFD30 and GFD30 versus GFD0 comparisons.
**Table S7:** Summary of GWAS datasets used for cross‐species genetic enrichment and spatial trait‐mapping analyses.

## Data Availability

The data supporting the findings of this study have been deposited in the CNGB Sequence Archive (CNSA) of the China National GeneBank DataBase (CNGBdb) under accession number CNP0010048 (https://db.cngb.org/data_resources/project/CNP0010048). The data that support the findings of this study have been deposited into Spatial Transcript Omics DataBase (STOmics DB) with accession number STT0000218 (https://db.cngb.org/stomics/project/STT0000218). The analysis scripts used in this study are publicly available at https://github.com/minwenl203-ctrl/Brain-gut-axis-in-germ-free-piglets. Supplementary materials (figures, tables, graphical abstract, slides, videos, Chinese translated version, and updated materials) may be found in the online DOI or iMeta Science http://www.imeta.science/.
