# Supplementary material for: Maternal microbiota shapes brain and neuroendocrine development in piglets
Source: Imeta. 2026 Oct 26;5(5):e70182. doi: 10.1002/imt2.70182 (PMC13702092; doi:10.1002/imt2.70182)
Supplement: Supplementary file 1 [file IMT2-5-e70182-s001.docx]

**Maternal microbiota shapes brain and neuroendocrine development in piglets**

Leli Wang¹˒²†, Chao Yan³†, Lei Li⁴†, Yuankun Deng²†, Kuo Long², Cimin Long¹, Yuanyuan Zhu¹, Meiqing Chen¹, Jialu Huang⁵, Jinghua Jiang⁶, Ziyi Lin⁴, Yunying Yao⁷, Tao Yang⁷, Wenbin Guo⁷, Hua Shao⁸, Zuohua Liu⁹, Jing Sun⁹, Yuchun Ding⁹, Liangpeng Ge⁹*, Mingzhu Yin⁸*, Yulong Yin¹˒²

†These authors contributed equally to this work.

Correspondence:

Leli Wang, leliwang@isa.ac.cn

Liangpeng Ge, geliangpeng1982@163.com

Mingzhu Yin, yinmingzhu2008@126.com

This file contains:

Supplementary Figures S1–S17

Supplementary Tables are provided in a separate Excel file.

**Figure S1. Regional distribution and functional characterization of the six major brain cell populations.** (A) UMAP visualizations of nuclei from AMY, HIP, HYP, mPFC, and BS, colored by major cell type. (B) UMAP visualizations of the integrated nuclei stratified by experimental group (GFD0, GFD30, and MMTD30). (C) Cell-type-specific expression patterns and functional enrichment of marker genes from the six major cell populations. Representative marker genes were grouped into six expression clusters (C1–C6) corresponding to microglia, endothelial cells, oligodendrocytes, astrocytes, OPCs, and neurons, respectively. The heatmap shows row-scaled expression (Z-score), and the line plots summarize the corresponding cell-type-specific expression patterns. Representative enriched GO Biological Process terms are shown for each cluster. Porcine experimental analyses were based on three independent piglets per experimental group (*n* = 3). Individual nuclei or cells were not treated as independent biological replicates.

Figure S2. Spatial transcriptomic characterization of major cell populations in the HYP. (A) ssDNA staining images of HYP tissue sections from GFD30 and MMTD30 piglets used for spatial transcriptomic analysis. (B) UMAP visualization of spatial transcriptomic profiles colored by predicted cell type. The integrated dataset is shown together with group-stratified visualizations for GFD30 and MMTD30. Six major predicted cell populations were represented: astrocytes, endothelial cells, microglia, neurons, oligodendrocytes, and OPCs. (C) Relative proportions of the predicted major cell populations in GFD30 and MMTD30 spatial transcriptomic datasets.

**Figure S3. Neuronal subtype markers, composition, developmental trajectories, and regional distribution.** (A) Feature plots showing representative markers of neuronal subtypes, including *GAD1* and *GAD2* for GABAergic neurons, *SLC17A7* and *CAMK2A* for glutamatergic neurons, and *DCX* and *SOX11* for immature neurons. (B) Relative proportions of GABAergic, glutamatergic, and immature neurons across the AMY, HIP, HYP, BS, and mPFC in GFD0, GFD30, and MMTD30 piglets. Cell-type proportions were analyzed at the animal level using beta-binomial models, followed by Benjamini–Hochberg FDR correction for multiple comparisons. Colored squares indicate neuronal subtypes showing significant differences between the indicated groups; *FDR < 0.05, **FDR < 0.01, and ***FDR < 0.001. (C) Differences in the proportions of GABAergic, glutamatergic, and immature neurons between MMTD30 and GFD30 piglets across the five brain regions. The x-axis represents the difference in cell proportion calculated as MMTD30 minus GFD30 in percentage points; positive values indicate higher proportions in MMTD30, whereas negative values indicate higher proportions in GFD30. Statistical significance was assessed using animal-level beta-binomial models followed by Benjamini–Hochberg FDR correction. *FDR < 0.05, **FDR < 0.01, and ***FDR < 0.001. (D) Pseudotime visualization of neuronal nuclei, colored by pseudotime (left) or experimental group (right). (E) Observed-to-expected (Ro/e) enrichment of GABAergic, glutamatergic, and immature neurons across five brain regions and three experimental groups. Dot size represents cell abundance as log_10_(count + 1), and color represents the Ro/e value. Ro/e values were used as descriptive measures of cell-distribution preference.

**Figure S4. Neuronal subtype-specific transcriptional responses across brain regions and experimental conditions.** (A)Neuronal-subtype-resolved differential gene-expression profiles between MMTD30 and GFD30 in the HIP, HYP, BS, and mPFC. Positive avg_log_2_FC values indicate higher expression in MMTD30, whereas negative values indicate higher expression in GFD30. Representative genes are labeled. (B) Differential gene-expression profiles of GABAergic, glutamatergic, and immature neurons between GFD30 and GFD0 across the AMY, HIP, HYP, BS, and mPFC. Positive avg_log_2_FC values indicate higher expression in GFD30, whereas negative values indicate higher expression in GFD0. Representative genes are labeled.

Figure S5. Immunofluorescence characterization and quantification of VGAT-positive GABAergic cells in the HYP. (A) VGAT immunofluorescence staining in HYP sections from GFD30 and MMTD30 piglets. VGAT is shown in green and nuclei were counterstained with DAPI in blue. Dashed boxes indicate regions of interest shown at higher magnification. Three independent piglets are shown for each group. (B) Quantification of VGAT-positive GABAergic cells per field in the HYP. Individual points represent independent biological replicates (*n* = 3 piglets per group). Data are presented as mean ± SEM. Statistical significance was assessed using a two-sided Student’s t-test.

**Figure S6. Region-specific remodeling of inferred neural–glial communication between GFD30 and MMTD30 piglets.** (A) CellChat analyses are shown separately for the AMY, HIP, HYP, BS, and mPFC. For each brain region, plots from left to right show: the numbers of inferred interactions and signaling pathways in GFD30 and MMTD30; differential intercellular communication networks inferred by CellChat between the MMTD30 and GFD30 conditions, encompassing astrocytes, GABAergic neurons, glutamatergic neurons, immature neurons, microglia, and oligodendrocytes. Upregulated interactions are indicated by red lines; downregulated interactions by blue lines; and comparison of inferred communication probabilities for ligand–receptor pairs shared between GFD30 and MMTD30. In probability scatter plots, the x‑ and y‑axes represent GFD30 and MMTD30 communication probabilities, respectively, and colors denote signaling pathways.

**Figure S7. Distribution, proportion changes, functional annotation, and region-specific transcriptional responses of major glial populations.** (A) Ternary representation of Ro/e enrichment patterns of major brain cell populations across GFD0, GFD30, and MMTD30. Colors indicate cell type, symbols indicate brain region, and point size represents Ro/e intensity. (B) Representative functional annotations of the six SCENIC co-regulatory modules (M1–M6). (C) Relative proportions of astrocytes, microglia, and oligodendrocytes across the AMY, HIP, HYP, BS, and mPFC in GFD0, GFD30, and MMTD30 piglets. Cell-type proportions were analyzed at the animal level using beta-binomial models, followed by Benjamini–Hochberg FDR correction for multiple comparisons. Colored squares indicate glial cell types showing significant differences between the indicated groups; *FDR < 0.05, **FDR < 0.01, and ***FDR < 0.001. (D) Ro/e enrichment of astrocytes, microglia, and oligodendrocytes across five brain regions and three experimental groups. Dot size represents cell abundance as log_10_(count + 1), and color indicates the Ro/e value. Ro/e values were used as descriptive measures of cell-distribution preference. (E) Differential gene-expression profiles of astrocytes, microglia, and oligodendrocytes between MMTD30 and GFD30 in the AMY, HIP, HYP, and mPFC. Positive avg_log2FC values indicate higher expression in MMTD30, whereas negative values indicate higher expression in GFD30. Representative genes are labeled.

**Figure S8. Developmental-stage-associated differential expression in major glial populations across brain regions.** (A) Differential gene-expression profiles of astrocytes, microglia, and oligodendrocytes between GFD30 and GFD0 across the AMY, HIP, HYP, BS, and mPFC. Positive avg_log_2_FC values indicate higher expression in GFD30, whereas negative values indicate higher expression in GFD0. Representative genes are labeled.

**Figure S9. Immunofluorescence characterization of IBA1-positive microglia in the HYP.** (A) IBA1 immunofluorescence staining in HYP sections from GFD30 and MMTD30 piglets. IBA1 is shown in green and nuclei were counterstained with DAPI in blue. Dashed boxes indicate regions of interest shown at higher magnification. Three independent piglets are shown for each group. (B) Quantification of mean IBA1 fluorescence intensity and IBA1-positive microglial soma area in the HYP. Individual points represent independent biological replicates (*n* = 3 piglets per group). Data are presented as mean ± SEM. Statistical significance was assessed using two-sided Student’s t-tests.

**Figure S10. Transcriptional heterogeneity and inferred developmental trajectories of astrocyte subclusters.** (A) Marker-gene expression patterns and representative functional annotations of six astrocyte subclusters (A1–A6). Marker genes were grouped into six corresponding expression patterns (C1–C6). The heatmap shows row-scaled expression (Z-score), and representative enriched biological processes are shown on the right. (B) UMAP visualization of A1–A6 in the integrated astrocyte dataset and stratified by GFD0, GFD30, and MMTD30. (C) Inferred trajectory of the six astrocyte subclusters, annotated by A1–A6. (D) Pseudotime distributions of astrocytes shown separately for GFD0, GFD30, and MMTD30, together with a combined visualization of the three experimental groups.

**Figure S11. Region- and subtype-specific astrocyte transcriptional responses associated with maternal microbiota transplantation.** (A) Differential gene-expression profiles of astrocyte subclusters A1–A6 between MMTD30 and GFD30 across the AMY, HIP, HYP, BS, and mPFC. Positive avg_log_2_FC values indicate higher expression in MMTD30, whereas negative values indicate higher expression in GFD30. Representative genes are labeled.

**Figure S12. *In silico* knockout analyses of *FOXO1* and *LMO1* in MMTD30 astrocytes.** (A) Regulatory network of genes predicted to respond to virtual *FOXO1* knockout. (B) GO Biological Process enrichment of genes predicted to respond to *FOXO1* knockout. (C) Regulatory network of genes predicted to respond to virtual *LMO1* knockout. (D) GO Biological Process enrichment of genes predicted to respond to *LMO1* knockout. In (B,D), dot size indicates gene count and color represents −log_10_(adjusted *p* value).

**Figure S13. Cell-type composition and inferred intercellular communication across the HPA axis.** (A) Relative cell-type composition across the HYP, PIT, and ADR in GFD0, GFD30, and MMTD30 piglets. Stacked bars indicate the proportions of annotated cell populations within each tissue and group. (B) CellChat-inferred intercellular communication networks among major PIT cell populations in GFD30 and MMTD30 piglets. Edge widths indicate the number of inferred interactions. (C) CellChat-inferred intercellular communication networks among major ADR cell populations in GFD30 and MMTD30 piglets. Edge widths indicate the number of inferred interactions.

**Figure S14. Cell-type-resolved transcriptional responses and HPA-related gene expression across the HPA axis.** (A) Differential gene-expression profiles of major cell populations in the HYP, PIT, and ADR between MMTD30 and GFD30 piglets. Positive avg_log_2_FC values indicate higher expression in MMTD30, whereas negative values indicate higher expression in GFD30. Representative upregulated and downregulated genes are labeled. (B) Differential gene-expression profiles of major cell populations in the HYP, PIT, and ADR between GFD30 and GFD0 piglets. Positive avg_log_2_FC values indicate higher expression in GFD30, whereas negative values indicate higher expression in GFD0. Representative upregulated and downregulated genes are labeled. (C) Heatmap showing expression patterns of selected HPA-axis-related genes across the HYP, PIT, and ADR in GFD30 and MMTD30 piglets. Colors indicate row-scaled Z-scores, and the bottom annotation indicates experimental group.

**Figure S15. Intestinal transcriptional variation, shared differential-expression patterns, and functional enrichment across developmental and microbial conditions.** (A) PCA of transcriptomic profiles from the duodenum, jejunum, ileum, cecum, and colon across GFD0, GFD30, and MMTD30 piglets. Colors indicate intestinal segments and symbols indicate experimental groups. PC1 and PC2 explain 34.91% and 18.26% of the variance, respectively. (B) Volcano plots showing DEGs between MMTD30 and GFD30 in the duodenum, ileum, cecum, and colon. Red and blue indicate upregulated and downregulated genes, respectively, whereas gray indicates genes without significant differential expression. The corresponding jejunal analysis is shown in Figure 5F. (C) Five-set Venn diagram showing seven genes consistently downregulated across the duodenum, jejunum, ileum, cecum, and colon in MMTD30 vs. GFD30. The seven shared genes are shown on the right. (D) STRING-based interaction network of the 50 genes consistently upregulated across all five intestinal segments in MMTD30 vs. GFD30. (E) GO Biological Process enrichment of DEGs in the GFD30 vs. GFD0 comparison across the five intestinal segments. The top five terms ranked by GeneRatio are shown for each segment; dot size indicates gene count and color indicates GeneRatio.

**Figure S16. Intestinal transcriptional responses across developmental and microbial conditions.** (A) Five-set Venn diagrams showing genes consistently upregulated (left) and downregulated (right) across the duodenum, jejunum, ileum, cecum, and colon in GFD30 vs. GFD0. Six shared upregulated genes and eight shared downregulated genes were identified, with their gene symbols listed on the right. (B) Volcano plots showing differential gene expression between GFD30 and GFD0 in the duodenum, jejunum, ileum, cecum, and colon. (C) GO Biological Process enrichment of GFD30 vs. GFD0 DEGs across the five intestinal segments. Dot size indicates gene count and color indicates GeneRatio. (D) Principal component analysis (PCA) of ileal transcriptomic profiles from GFD30 and MMTD30 piglets. PC1 and PC2 explained 47.19% and 19.22% of the total variance, respectively. Colors indicate experimental groups.

**Figure S17. gsMAP-based analysis of major-cell-lineage and cluster-level associations with human complex traits in the porcine brain.** (A) Heatmaps from gsMAP analysis showing −log_10_(p Cauchy) values for trait-associated signals across eight clusters in GFD30 (upper) and MMTD30 (lower). Rows represent clusters and columns represent the analyzed traits. Higher −log_10_(p Cauchy) values indicate stronger statistical evidence for the corresponding cluster–trait association. EA2, educational attainment; IQ, intelligence quotient.
